# Role of HIV-1 Tat Protein Interactions with Host Receptors in HIV Infection and Pathogenesis

**DOI:** 10.3390/ijms25031704

**Published:** 2024-01-30

**Authors:** Aurelio Cafaro, Ivan Schietroma, Leonardo Sernicola, Roberto Belli, Massimo Campagna, Flavia Mancini, Stefania Farcomeni, Maria Rosaria Pavone-Cossut, Alessandra Borsetti, Paolo Monini, Barbara Ensoli

**Affiliations:** National HIV/AIDS Research Center, Istituto Superiore di Sanità, 00161 Rome, Italy; ivan.schietroma@iss.it (I.S.); leonardo.sernicola@iss.it (L.S.); roberto.belli@iss.it (R.B.); massimo.campagna@iss.it (M.C.); flavia.mancini@iss.it (F.M.); stefania.farcomeni@iss.it (S.F.); mariarosaria.pavonecossut@iss.it (M.R.P.-C.); alessandra.borsetti@iss.it (A.B.); paolo.monini@iss.it (P.M.)

**Keywords:** HIV-1 Tat protein, extracellular Tat protein, HIV-1 infection, HIV-1 pathogenesis, HIV-1 Env, HIV-1 Tat/Env complex, HIV vaccine, HIV preventative vaccine, HIV therapeutic vaccine, HIV functional cure

## Abstract

Each time the virus starts a new round of expression/replication, even under effective antiretroviral therapy (ART), the transactivator of viral transcription Tat is one of the first HIV-1 protein to be produced, as it is strictly required for HIV replication and spreading. At this stage, most of the Tat protein exits infected cells, accumulates in the extracellular matrix and exerts profound effects on both the virus and neighbor cells, mostly of the innate and adaptive immune systems. Through these effects, extracellular Tat contributes to the acquisition of infection, spreading and progression to AIDS in untreated patients, or to non-AIDS co-morbidities in ART-treated individuals, who experience inflammation and immune activation despite virus suppression. Here, we review the role of extracellular Tat in both the virus life cycle and on cells of the innate and adaptive immune system, and we provide epidemiological and experimental evidence of the importance of targeting Tat to block residual HIV expression and replication. Finally, we briefly review vaccine studies showing that a therapeutic Tat vaccine intensifies ART, while its inclusion in a preventative vaccine may blunt escape from neutralizing antibodies and block early events in HIV acquisition.

## 1. Introduction

The introduction in the late 1990s of the combination antiretroviral therapy (cART) has dramatically changed the course of the infection, as virtually all successfully treated individuals do not progress to AIDS (acquired immune deficiency syndrome) [1] nor transmit infection [2]. However, cART suppresses virus replication and spreading but it does not eliminate latently infected cells, and residual HIV protein expression and virus production are still detected upon sporadic virus reactivation [3,4], particularly in the gut and central nervous system [5,6].

Of note, HIV-1 replication requires Tat, the trans-activator of transcription, whose main role in the HIV-1 life cycle is to promote gene expression and virus production [7]. In fact, in the absence of Tat, virtually no productive infection occurs. Interestingly, in the acute infection about two-thirds of the Tat protein is released extracellularly [8,9,10,11,12] and exerts effects on both the virus and many cell types key to HIV acquisition, spreading, pathogenesis and progression to disease.

Thus, targeting Tat it is important in several respects: to prevent the establishment of infection and, in people living with HIV (PLWH), to reduce the burden of the residual disease (chronic inflammation and immune activation, early aging) commonly observed in individuals on suppressive cART [13] and responsible for the reduced quality of life and life expectancy [1]. Finally, targeting Tat may be critical to eradicating HIV.

Here, we review the role of Tat in HIV-1 infection and pathogenesis with a particular focus on the role the extracellular protein (eTat), and we propose that targeting Tat in preventative and therapeutic vaccine approaches may be critical for vaccine efficacy.

## 2. The HIV-1 Tat Protein

Tat is a 14–16 kD HIV regulatory protein whose main role in the HIV life cycle is to promote virus transcription, and primarily transcript elongation. In fact, Tat is prominently known for its role in relieving RNA polymerase II from pause, thus promoting elongation, a key step leading to the completion of HIV gene transcription [14]. However, Tat is also required to initiate reverse transcription (RT) [7], to increase the rate of transcription [7] and to contribute to splicing regulation [15,16].

Tat is generated in two forms through alternative splicing. The first form is encoded by the multiply spliced two-exon transcript and varies in length between 86 and 101 amino acids, depending on the viral isolate, whereas the other form is encoded by a singly spliced one-exon transcript and is 72 amino acids long. Both Tat variants transactivate the LTR efficiently, but the two-exon Tat appears to exert additional effects on the infected cell, such as altering cytoskeleton structure and function [17], delaying Fas-mediated apoptosis [18], and reducing the triggering of innate and adaptive immune responses by downregulating expression of interferon-stimulated genes and MHC class-I and II molecules in antigen-presenting cells [19,20,21]. Here, unless differently stated, Tat refers to the 86 amino acids (aa)-long two-exon Tat protein, which is the most commonly used form of Tat [22].

**Figure 1:**
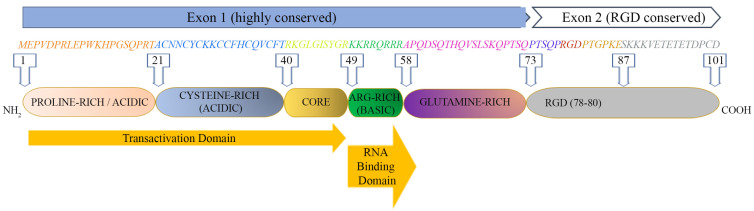
HIV-1 Tat sequence (HXBc2 strain) and functional domains. See text for Tat domains’ detailed description.

The Tat protein is largely unstructured and contains six functional domains (Figure 1). These features make Tat capable of interacting with many molecules, as it can easily adapt to a molecule displaying one or more complementary domains or motifs.

The first domain (1–21 aa) at the N-terminus is an acidic proline-rich region and has a conserved Trp 11 that is critical to stabilize Tat binding to the inner leaf of the cell membrane [12]; the second domain (residues 22–37) has seven cysteines that are rather well conserved at positions 22, 25, 27, 30, 31, 34 and 37; six of them may establish intramolecular bonds, while the seventh cysteine is believed to mediate intermolecular bridging; the third domain (core, residues 38–48) contains two residues of a conserved four-amino-acid subdomain (36–39) reported to bind tubulin/microtubules through a microtubule-associated protein, LIS1 [23], leading to the alteration of microtubule dynamics and activation of a mitochondria-dependent apoptotic pathway [24,25]. Overall, the N-terminal half (1–48 aa, region I–III) of Tat is highly conserved, consistent with the critical role for activating the transcription of HIV genomic DNA due to the cysteine-rich motif (region II), required for dimerization, protein structure stabilization and metal binding, and the hydrophobic core motif (region III) required for binding to the CDK9-associated C-type cyclin [26] and to the transactivation response RNA element (TAR) of the newly transcribed HIV genomic RNA [27,28].

The C-terminal half (49–72 aa) of Tat contains two more regions: region IV (residues 49–55) is a conserved basic domain important for localizing Tat to the nucleus, binding of Tat to TAR and the internalization of the Tat protein into bystander cells by its interaction with surface proteins such as heparan sulfate proteoglycans (HSPG) [29,30]. Of note, a peptide corresponding to the basic region is currently exploited as a penetratin or transducing molecule to intracellularly deliver numerous cargos (proteins, DNA, RNA), underscoring its ability to cross membranes [31]. Residues 59–72 encompass the glutamine-rich domain (region V), which is less conserved and participates in the interaction of Tat with TAR and in Tat-mediated apoptosis [32].

Although more variable than Exon 1, most Exon 2 sequences (region VI) contain an arginine-glycine-aspartic acid (RGD) motif (78–80 aa) which is important for the Tat interaction with the RGD-binding integrins αvβ1, αvβ3, α5β1 [33,34]. Whether and to what extent Tat binds the other 5 RGD binding integrins remains to be investigated. Although not required, the second exon of Tat contributes to optimal virus replication in T cells and macrophages [35,36]. In T cells, Tat101 delayed FasL-mediated apoptosis, prolonging HIV-1 replication [18], and altered the function and distribution of mitochondria [37]. In myeloid cells, the two-exon protein has been reported to reduce the innate immunity activation observed with one-exon Tat [19].

Tat is expressed very early upon infection, even before virus integration [38]. Indeed, accumulating evidence indicates that Tat is incorporated into HIV-1 virions [39,40], thus priming both intra-virion and post-entry reverse transcription [41] and activating virus gene expression even prior to HIV gene expression [40].

## 3. Mechanism and Kinetics of the Extracellular Release of Tat (eTat)

The majority (about 65%) of the Tat protein produced by the infected cell is released extracellularly in the absence of cell death or cell permeability changes mainly by a leaderless secretory pathway similar to that used by basic fibroblast growth factor (FGF)2 to exit cells [8,10,11,12,42]. However, none of the unconventional secretory pathways used by FGF or interleukin (IL)-1 β are involved, as Tat appears to traffic to the plasma membrane without the contribution of intracellular intermediates [12]. In particular, the conserved Arg49, Lys50 and Lys51 (RKK motif) of Tat appears to bind the fraction of phosphatidylinositol-(4,5)-bisphosphate [PI(4,5)P2] located at the plasma membrane [12]. This binding is strengthened by the insertion of Tat Trp11 into a hydrophobic cleft of the inner membrane [12]. An additional interaction between the basic region (RRQRRR) of Tat and phosphatidylserine has also been reported very recently [43]. These interactions may affect several biological processes in which PI(4,5)P2 is involved, such as clathrin-mediated endocytosis [44], phagocytosis [45,46] or exocytosis [47]. Plasma-membrane-bound Tat is then released extracellularly by exocytosis [11], with still incompletely understood mechanism(s) (reviewed in [42]). Recent evidence indicates that Tat is also released extracellularly within exosomes, which appear to be enriched with small noncoding RNAs containing TAR and their derivative TAR miRNA [48], which, in turn, have been reported to promote, respectively, inflammation and tumorigenesis, two hallmarks of the residual disease observed in subjects on long-term suppressive cART [49,50,51]. Of note, extracellular Tat (eTat) crosses the blood–brain barrier and promotes the central nervous system (CNS) inflammation and T-cell activation [52], which persist despite treatment intensification with CNS-penetrating ART [53].

Upon release, eTat binds heparan sulphate proteoglycans (HSPG) of the extracellular matrix (ECM) and is detected in the tissues of infected individuals [8,9,11,52,54,55,56,57]. eTat is biologically active and exerts activities key for the acquisition of infection, virus reactivation and HIV disease maintenance in cART-treated individuals [9,10,11,12,38,55,56,57,58,59,60,61,62,63,64,65,66,67,68].

Up to 40 ng/mL (4 nM) of eTat has been detected in biological fluids, but these amounts are probably underestimated, as eTat accumulates in tissues, bound to the HSPG of the extracellular matrix (ECM) in a biologically active form [8,52,54,55,56,57]. eTat is also known to be taken up by uninfected cells, including T lymphocytes, macrophages and neurosecretory cells and to accumulate at the plasma membrane, where it stably binds to phosphatidylinositol (4,5) bisphosphate (PI(4,5)P2), thus interfering with the (PI(4,5)P2)-mediated functions, such as neurosecretion, phagocytosis and cardiac muscle repolarization [69]. For stable binding to (PI(4,5)P2), interaction of the Tatcys31 with cyclophilin A (CypA) and Tat palmitoylation by HHD20 are required [69]. Intriguingly, the Indian clade C Tat displays a naturally occurring cysteine 31 substitution that abolishes (PI(4,5)P2) sequestration by Tat, possibly contributing to the apparent lower pathogenicity of clade C Tat as compared to clade B [69]. In sharp contrast, Tat is efficiently released in productively infected cells. CypA binds the newly produced HIV-1 Gag protein, it is incorporated into the capsid and it is released from the cell within the budding virus [40]. As a result, intracellular CypA is depleted and Tat palmitoylation cannot occur [69].

## 4. Role of eTat in HIV Acquisition, Dissemination and Reservoir Formation

### 4.1. HIV Acquisition

HIV is mostly acquired through sexual intercourse, with the rate of acquisition being extremely low, which varies according to the type of unprotected sex (vaginal/anal/oral; insertive/receptive [70,71,72,73], and it is strongly favored by pre-existing genital infections [74] and viral load [75,76,77,78]. Thus, mucosae represent a strong barrier to the acquisition of HIV (reviewed in [79]), which must undergo a major selection to overcome the mucosal barrier [80], resulting in the systemic spreading of founder viruses characterized by Env with a “short” V1/V2 loop [81].

In this context, our data indicate that Tat binds native trimeric Env on HIV-1 virions to form a novel cell entry complex (the Tat/Env complex), enabling the virus to enter dendritic cells (DCs) through an integrin-mediated endocytic pathway alternative to the canonical endocytic pathway mediated by C-type lectin receptors [82]. Tat-mediated entry in DCs leads to the enhancement of HIV infection in these cells [82], which are key for HIV acquisition at the mucosal portal of entry. The Env V3 loop is a main Tat binding determinant, as a cyclic (but not linear) peptide encompassing the V3 loop was shown to bind recombinant Tat, and V3 loop deletion abrogates the capability of Tat to direct Env on the integrin receptors [82]. In this regard, the Env V1/V2 loop is known to engage and occlude the V3 loop at the Env trimer apex [83], and our studies indicate that V1/V2 loop shortening dramatically increases the stability of the Tat/Env complex [82]. These data might explain why V1/V2 shortening is a common feature of the founder viruses emerging at the mucosal portal of entry.

Since the Tat/Env complex drives HIV to the DCs integrin receptors, anti-Env Abs blocking the Env interaction with C-type lectin receptors become ineffective at preventing HIV-1 entry in these cells [82]. However, entry is blocked by the addition of anti-Tat Abs [82]. Indeed, the immunization of monkeys with the Tat/Env complex led to infection containment upon intrarectal challenge with a pathogenic simian-HIV chimeric virus (SHIV), preventing virus spreading beyond the rectum [82]. Taken together, these data suggest that eTat plays a key role also in HIV-1 acquisition, providing the rationale to also include it in preventative vaccine approaches in association with Env.

### 4.2. HIV Dissemination

As Tat is required for HIV gene expression, it is apparent it plays a critical role in promoting virus replication and, ultimately, dissemination. Indeed, seroreversion of the antibody response to HIV, a bona fide proxy of virus remission or eradication, was reported in a small cohort of 23 women from Gabon infected with a Tat in which the cysteine in position 22 was replaced by a serine (Tat Oyi) making its transactivation silent [84]. Similarly, in the cART era, progressive accumulation over time of proviruses defective for replication [85] and/or for gene expression (i.e., containing solo LTRs) [86] was found. This is probably the result of the combined pressure exerted by cART and by a sufficiently restored immune response against HIV. In this scenario, only silent proviruses lacking Tat, as solo LTRs, are destined to persist.

### 4.3. Establishment and Maintenance of Latent Virus Reservoirs

Despite cART effectiveness, a low-level intermittent residual plasma viremia (<50 copies per mL), as well as viral “blips” (50–1000 copies/mL), persist, predict virus rebound and are believed to be at the origin of persistent immune activation, residual disease and the onset of comorbidities in treated patients (reviewed in [13]). In fact, HIV gene expression and viral production are sporadically resumed in HIV latently infected cells constituting the HIV reservoirs [3,4].

Furthermore, HIV gene expression it is not halted by cART, whereas residual virus replication, driven by low drug penetration in lymphoid tissue compartments [87,88] and drug-resistant cell-to-cell transmission modalities [89], may occur [90]. Of importance, while Tat mRNA accumulates in the nuclei of resting CD4 T cells from peripheral blood and cannot support HIV protein expression [91], resting CD4 T cells from lymphoid tissues do express positive transcription elongation factor (PTEF)b1 and support HIV-1 gene expression and replication, allowing HIV acquisition at the portal of entry [92] and likely contributing to residual virus replication and reservoir replenishment.

Intracellular HIV-1 Tat plays a key role in virus reservoir establishment and maintenance. In fact, Tat expression enhances stochastic fluctuations of the basal HIV transcriptional machinery driven by host transcriptional factors [54,93,94]. This, in turn, drives Tat expression itself into stochastic oscillations around the threshold of virus transcriptional extinction. Consequently, the Tat positive transcriptional feedback loop is pivotal for fate decision-making between HIV productive and latent infection [54,93,94]. The stochastic features of the Tat circuitry may in part explain the failure of “shock and kill” strategies to eradicate HIV based on the deterministic reversion of HIV latency through latency-reversing agents [95]. In contrast, agents inhibiting the Tat transcriptional loop effectively block the reactivation of latent HIV, which has led to the “block and lock” strategy for a permanent shut-off of HIV reservoirs [96].

Not surprisingly, Tat is produced and released in treated patients [52,97,98], pointing to a role for eTat in HIV reservoir dynamics. As already mentioned, eTat crosses the blood–brain barrier and chemoattracts monocytes/macrophages and T cells, promoting inflammation, the permissivity of resting T cells and T-cell activation, [52]. Thus, eTat promotes the establishment and maintenance of an HIV reservoir in the CNS, which is relatively insensitive to immune control and ART [53]. In this context, several lines of evidence also suggest that eTat plays a key role in the establishment and maintenance of the memory CD4 T cell reservoir. In fact, eTat promotes the activation and differentiation of naïve CD4 T cells towards the effector-memory phenotype [99], thus increasing the frequency of cells transitioning from the activated to the resting state, which, in turn, is associated with HIV latent infection [100]. Further, it upregulates anti-apoptotic genes, particularly Bcl-2, in CD4 T cells, promoting their survival [101]. Taken together, it is apparent that eTat is pivotal not only in HIV acquisition and spreading, but also in the establishment and maintenance of reservoirs.

Extensive evidence of the role of intracellular Tat in HIV reservoirs has been reviewed elsewhere [102].

## 5. eTat Interaction with Host Molecules

Being unstructured and with several functional domains, Tat may interact with many molecules. Here, we briefly describe those that are most likely to contribute to the chronic inflammation and immune dysregulation evidenced in virologically suppressed PLWH, consistent with the notion that residual HIV gene expression and viremia depend on Tat protein expression.

### 5.1. Engagement of RGD-Binding Integrins

The Tat protein from HIV-1 Clade B and D viruses contains at its carboxy-terminus the Arg-Gly-Asp (RGD) tripeptide motif [33,65,103] present on several ECM proteins, including fibronectin, vitronectin, thrombospondin, laminin, tenascin and others. This motif represents the recognition domain for RGD-binding integrins, a sub-set of which are recognized by eTat (αvβ3, αvβ5 and α5β1) and are highly expressed by activated endothelial cells (ECs) [104,105,106,107,108,109,110] and DCs [82,109,110,111,112]. The role exerted by the interaction of eTat with DC integrin receptors in virus acquisition at the mucosal portal of entry has been reviewed in Section 4.1. In this section, we review studies pointing to the relevance of this interaction in HIV-related EC dysfunction and AIDS-associated Kaposi’s sarcoma pathogenesis, as well as for the innate immune responses driven by DCs in HIV-infection.

Owing to the continuous exposure to IC, ECs are chronically activated in HIV infection, and undergo numerous phenotypic and functional alterations characteristic of endothelial dysfunction, which are associated with the increased incidence of cardiovascular diseases observed in people living with HIV. Several studies indicate that eTat may play a key role in these processes. In particular, both the expression and/or function of αvβ3 and α5β1 integrins in ECs are increased by the inflammatory cytokines (IC) augmented in HIV-infected individuals, particularly IFN-γ, TNF-α and IL-1β, even in people on effective antiretroviral treatment [113,114]. These IC render ECs responsive to eTat. In fact, by targeting the αvβ3 and α5β1 integrins, the RGD motif of eTat mimics the effects of ECM molecules, leading to EC growth, migration and invasion, and provides ECs with the adhesion signal required for growth in response to mitogens at sites of ECM remodeling [34,115,116,117,118,119]. The basic region of eTat cooperates with the RGD motif in these effects by retrieving FGF2 bound to HSPG in a soluble form that increases αvβ3 expression and mediates eTat-induced vascular cell growth [105]. Our recent data also indicate that eTat is internalized very efficiently by activated ECs through the αvβ3, αvβ5 and α5β1 integrins, leading to events supporting the production and release of infectious virus by these otherwise poorly/non-susceptible cells [107]. Since Tat is produced and released even during cART [52,98], all of the above data indicate that the interaction between eTat and the αvβ3, αvβ5 and α5β1 integrins may exacerbate endothelial dysfunction while increasing the risk of cardiovascular diseases in treated patients, with implications for novel Tat-based therapeutic approaches.

In this context, several studies have shown that eTat is involved in the pathogenesis of Kaposi’s sarcoma (KS), an angio-proliferative tumor arising with high frequency in HIV infection (AIDS-KS) in a background of CD8 T cells activation, increased expression of Th1 cytokines, particularly IFN-γ, and ECs activation and dysfunction [120,121]. KS starts as a reactive process, characterized by the appearance of granulation-like tissue infiltrated by reactive lympho-monocytes and macrophages, and it evolves toward a multifocal plaque or nodular tumor showing intense neoangiogenesis, oedema and the growth of spindle-shaped cells (KS spindle cells, KSC), believed to be the tumor cells of KS. KSC are a heterogeneous population of cells of vascular and lymphatic endothelial origin, mixed with cells expressing markers of the so-called endothelial macrophages [121]. eTat induces the adhesion growth, migration and invasion of cultured KSC [8,10,34,105,122] and synergizes with FGF2 in the induction of KS-like lesions in nude mice [9]. The interaction of the RGD motif of Tat with the αvβ3 and α5β1 integrins is key for these actions of eTat; in fact, the development of KS-like lesions in mice inoculated with Tat and FGF2 is inhibited by RGD peptides [122]. Moreover, transgenic mice expressing HIV-1 Tat develop larger and more severe KS-like lesions compared with mice transgenic for an RGD-deleted Tat [123], further emphasizing the role of the interaction of eTat with RGD-binding integrins in this AIDS-associated tumor.

Other data from our studies also indicate that the αvβ3, αvβ5 and α5β1 integrins mediate the efficient internalization of eTat not only in ECs but also in DCs [66,82]. The entry of eTat in DCs induces their maturation, up-regulates MHC and costimulatory molecules and induces Th1 cytokines and beta-chemokines production, leading to a polarization of the immune response toward a Th1 pattern [66,67]. These effects are due to the transactivation activity of Tat, as they are not observed with the transactivation-silent Tatcys22 mutant, which enters DCs with comparable efficiency as compared to native Tat [66]. Accordingly, native Tat and Tatcys22 induced a predominant Th1 or Th2 response, respectively, in inoculated cynomolgus monkeys [67].

In contrast to ECs, RGD-binding integrins are not required for the locomotion/migration of DCs and macrophages [124,125]. Rather, αvβ3 and/or αvβ5 expressed by DCs and macrophages act in cooperation with other receptors in the recognition and engulfment of apoptotic bodies, leading to phagocytic clearance of dying/dead cells (efferocytosis) [126]. If the dying cell is infected by pathogens, this results in the cross-presentation to CD8 T cells of foreign antigens [108,111,126,127,128,129] and, for HIV-infected cells, in the efficient infection of the engulfing macrophage [130]. These aspects would deserve studies aimed at investigating the role of the interaction of eTat with integrin receptors in these processes.

### 5.2. eTat Binds and Inhibits CD26

CD26 is a 110 kDa glycoprotein endowed with dipeptidyl peptidase IV (DPPIV) activity. This ectoenzyme cleaves N-terminal dipeptides from polypeptides with proline, hydroxy-proline and, with less efficiency, alanine in the penultimate position. CD26 is a type II integral membrane glycoprotein displayed in the form of a homodimer on the cell surface of many cell types, including myeloid and lymphoid cells, and in a soluble form in several biological fluids [131]. On human T cells, CD26 exerts a co-stimulatory role via its ability to bind and inhibit adenosine deaminase (ADA), association with CD45 and cleavage and modulation of the activity of many inflammatory cytokines and chemokines, including CCL5 and CXCL12, natural ligands of the HIV-1 co-receptors CCR5 and CXCR4, respectively [131,132]. In general, the inhibition of CD26 activities results in immunosuppression, potentiated by transforming growth factor (TGF)b1, which is induced by CD26 inhibition [133,134]. In HIV-1 infection, low CD26 levels and enzymatic activity and increased TGFb1 plasma levels have been reported to correlate with progression to disease [135,136]. eTat was reported to bind CD26 and to inhibit its enzymatic activity [137], leading to T-cell anergy [138]. The N-terminus of Tat (Tat 1–9) was sufficient for the binding, while the inhibition of the enzymatic activity mapped to the N-terminal tripeptide (Tat 1–3) [139]. As eTat was also reported to induce TGFb1 [140], CD26 inhibition likely contributes to the dysregulation observed in PLWH progressing to disease [135]. Indeed, the addition of soluble CD26 was shown to revert the T-cell anergy of PBMC from PLWH, likely due to the blocking of eTat [141].

### 5.3. Binding and Triggering of Inflammatory Chemokine Receptors

Initial reports indicated that eTat induces monocyte cell adhesion to ECs [142], as well as the migration and invasion of monocytes [143]. It was later established that this was due to molecular mimicry between Tat and several CC inflammatory chemokines, and the cysteine-rich and core regions of Tat were identified as the key domains mediating this effect [144]. In particular, eTat was found to compete with CCL2, CCL7 and CCL11 for binding to CCR2 and CCR3 and to act as an agonist of these chemokines [145]. In contrast, eTat acts as an antagonist of CXCL12, possibly contributing to the observed prevalence of the transmission of R5-tropic strains [54]. Thus, eTat released by acutely infected cells chemoattracts monocytes/macrophages, DCs and lymphocytes, favoring HIV spreading. Notably, eTat also induces marked increases in CCR3, CXCR4 and CCR5 HIV co-receptors on monocyte/macrophages and of CXCR4 on T lymphocytes, enhancing HIV infectivity in both cell types and expanding the tropism of X4-strains to monocyte/macrophages [146]. In particular, clade B but not clade C eTat promotes the entry of X4-tropic HIV strains in resting CD4 T cells [147] and the early establishment of reservoirs [148], and it correlates with HIV-1 viral load, disease progression and dementia (reviewed in [149]).

eTat binding to CCR2 and CCR3 also activates target cells and triggers the release of inflammatory cytokines and chemokines, further fueling HIV infection and dysregulating the host homeostasis. Indeed, CCR2, which is expressed constitutively in monocytes and downregulated in macrophages, is induced in inflammatory conditions in subsets of basophils, NK cells and T cells [150]. Similarly, levels of both CCL2, the main ligand of CCR2, and CCR2, the most potent receptor for myelomonocytic cell recruitment to acutely inflamed sites [151], are increased and correlate with disease progression in PLWH [149,152], characterized by the occurrence of cardiovascular diseases (CVD), HIV-associated neurological disorders (HAND), bone frailty, metabolic disorders, renal and liver failure and cancer [153,154]. Of interest, the overactivation of the CCL2–CCR2 axis has been implicated in the pathogenesis of all of these morbidities [155,156,157,158,159,160,161]. The protective role attributed to antibodies directed against the cysteine-rich domain of Tat is consistent with this view [162].

Thus, by inducing chemokine receptors’ expression and by mimicking chemokines, eTat generates a module capable of increasing the susceptibility to virus infection also in relatively HIV-resistant contexts, such as at the portal of entry [80].

### 5.4. Binding and Activation of TLR4-MD2-CD14

A hallmark of HIV-1 infection is the abnormal activation of inflammation and of the immune system, which persists at a low grade even in PLWH on effective cART [163]. Residual HIV gene expression and replication have been implicated, and Tat is a prominent suspect, as HIV gene expression occurs only in the presence of the Tat protein [7]. Indeed, eTat has been reported to stimulate the production of proinflammatory (IL-1β, IL-6, IL-8, IFN-γ, TNF-α) and anti-inflammatory (IL-10) cytokines in human monocytes/macrophages and of IL-6, IL-8 and IDO in monocyte-derived dendritic cells (MDDCs) from both healthy and HIV-1 infected patients [164]. This effect of eTat was abolished by anti-Toll-like receptor 4 (TLR4) blocking antibodies or soluble recombinant TLR4-MD2 as a decoy receptor and led to the discovery that the N-terminus of eTat binds with high affinity to the TLR4-MD2-CD14 complex and activates the NF-κB pathway [165]. In MDDCs, exposure to eTat also induced the expression of PD-L1 through an indirect mechanism involving TNF-α and TLR4 pathways [166]. Induction of IDO and PD-L1 by eTat hampered the MDDC-driven activation of T cells [164,166]. Thus, through TLR4 engagement eTat appears to promote the inflammation and dysregulation of the immune response observed in virologically suppressed PLWH [163]. Notably, this may account for the inherent adjuvanticity of the Tat protein administered as a vaccine in mice, monkeys and humans [97,167,168,169,170,171,172,173,174].

### 5.5. Binding to LRP1 (CD91)

The low-density lipoprotein receptor-related protein (LRP1, CD91) is a large multifunctional receptor with a variety of structurally diverse ligands. As a scavenger receptor, it clears fibrinolytic system components, complement factors, lipoproteins, inflammatory cytokines cellular and pathogens debris from the circulation [175]. LRP1 modulates inflammatory responses and has anti-inflammatory effects by inhibiting pro-inflammatory factors such as tissue necrosis factor (TNF-α), interleukin (IL)-1, IL-6, IL-10 or type 1 interferons [175,176]. It is ubiquitously expressed in many cell types, including myeloid and lymphoid cells [176]. In particular, it is expressed at high levels in monocytes and macrophages, followed by DCs, whereas the expression is low in T and B lymphocytes and upregulated in T cells upon activation [176]. In macrophages, CD91 mediates efferocytosis and the suppression of inflammatory cytokines production, while in T cells it regulates adhesion, trafficking and activation [177,178]. The increased expression recorded on monocytes from long-term nonprogressors (LTNP) and multiply exposed uninfected individuals (MEU) would suggest a protective role, possibly due to the prevalent anti-inflammatory and immunosuppressive function of LRP1 [179,180].

eTat was reported to bind CD91 and to compete out the many ligands of LRP1 [181], thus interfering with its physiological activities. However, the scenario is more complex in light of the interactions eTat has with thrombospondin and calreticulin, both key partners of LRP1 in the regulation of T-cell functions [177,178], and the control CD26 exerts on thrombospondin and CD91 membrane expression and function [182].

## 6. Interaction of eTat with Host Receptors: A Way to Immune Dysregulation

**Figure 2:**
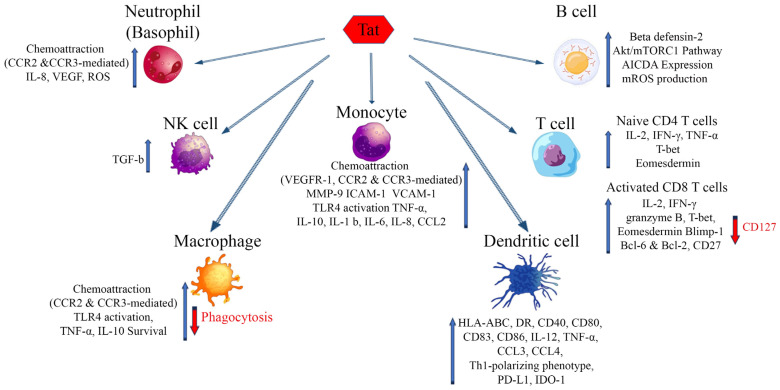
Effects of extracellular Tat on cells that are key players of the innate and adaptative immune response. Arrows indicate increase (blue) or decrease (red) of the markers shown. AICDA: Activation-induced cytidine deaminase; Bcl-2, -6: B-cell lymphoma 2, 6; Blimp-1: B lymphocyte-induced maturation protein-1; CCR2: C-C chemokine receptor type 2; CCR3: C-C chemokine receptor type 3; CCL-2, -3, -4: chemokine (C-C motif) ligand 2, 3, 4; HLA-ABC: Human leukocyte antigens A, B, C; HLA-DR: HLA class II cell surface receptor; ICAM-1: Intercellular Adhesion Molecule 1; IDO-1: Indoleamine 2,3-dioxygenase; IL: Interleukin; IFN-γ: Interferon gamma; MMP-9: Matrix metalloproteinase-9; mROS: mitochondrial reactive oxygen species; mTORC1: mammalian target of rapamycin complex 1; PD-L1: Programmed death-ligand 1; T-bet: T-box transcription factor TBX21; VEGF: Vascular endothelial growth factor; VEGFR-1: Vascular endothelial growth factor receptor 1; VCAM-1: Vascular cell adhesion protein 1.

HIV-1 infection is characterized by a generalized dysregulation of innate and adaptive immunity mediated by myeloid and lymphoid cells, respectively [13]. The molecules mentioned above and binding eTat are expressed mostly on myeloid and to a lesser extent lymphoid cells, and their engagement by eTat has been reported to alter their regulation and function, thus contributing to the sustained inflammation and immune dysregulation characterizing disease progression (Figure 2) [13]. The overall outcome is a dysregulated immune activation and suppression, driving on the one hand HIV dissemination and latency, on the other hand, immune deficiency and the occurrence of co-morbidities. Of note, this is further compounded by the reported dichotomous role Tat has on cell survival and death. In general, in infected cells (intracellular) Tat appears to promote cell survival by inducing Bcl2 and blocking Bim, whereas in uninfected cells eTat promotes apoptosis [24,183,184].

## 7. Antibodies to Tat Are Associated with No or Delayed Progression to Disease

Anti-Tat antibodies (Abs) are infrequent in PLWH [185,186]. Of note, an inverse relationship between progression to disease (associated with p24 antigenemia, plasma VL and CD4 T cell loss) and anti-Tat seropositivity was noticed, suggesting a protective role of anti-Tat Ab [187,188,189,190]. Indeed, a significantly lower risk of progression to disease was observed in a cohort of 252 HIV-1 seroconverters followed for up to 14 years (median follow-up time: 7.2 years) [191]. Similarly, the time to cART initiation differed markedly according to the anti-Tat Ab serostatus in a subsequent prospective observational (OBS) study (NCT01029548) (Table 1) [192]. In fact, of the 61 asymptomatic individuals naive to cART and followed for up to 42 months (median follow-up time: 24 months), all of those seronegative for anti-Tat Abs had to start cART by 17 months, while none of the 20 (32.8%) subjects with a broad (all the three classes of immunoglobulins) and durable anti-Tat Ab response had to do so by the end of the study (Table 1) [192]. Further, the anti-Tat serostatus also affects the response to cART initiation. A faster (>3 times) and stronger (persistently undetectable VL) response to therapy was observed in anti-Tat Ab positive subjects as compared to anti-Tat Ab negative individuals (*p* < 0.0001, log-rank test (OBS-IFO) (Table 1) [193]. In individuals on long-term ART, anti-Tat immunity was associated with higher nadir CD4 T cell counts, long-lasting CD4 T cell recovery and the control of low-level viremia [194]. Interestingly, the anti-Tat Ab level was associated with the control of very-low-level viremia (≤40 copies/mL) whereas the occurrence of higher (>40 copies/mL) viral load was not affected by the anti-Tat serostatus (Table 1) [195]. It will be of interest to determine whether inducing or boosting anti-Tat immune responses improves virus control in individuals on long-term ART. Thus, strategies aimed at inducing Abs capable of neutralizing the biological activity of eTat should be pursued to halt the eTat-mediated maintenance of HIV-1 reservoirs and reduce inflammation and immune activation and dysregulation.

## 8. Strategies to Neutralize eTat in Preventative and Therapeutic Vaccine Approaches

Vaccination is undoubtedly the most cost-effective intervention to curb the HIV pandemic, both in the preventative and therapeutic setting. Based on the epidemiological evidence and experimental data indicating a protective role for anti-Tat immunity, in particular of anti-Tat Abs, the development of vaccines based on Tat was undertaken.

### 8.1. Pre-Clinical and Early (Phase I) Clinical Development

The Tat vaccine underwent an extensive (9 studies employing 112 Mauritian cynomolgus monkeys) preclinical evaluation in nonhuman primates [169,170,196,197]. Overall, Tat vaccination reduced infection acquisition and contained acute CD4 T cell loss in both acute and chronic infection [170].

Based on these results, preventative (ISS P-001, ClinicalTrials.gov Identifier: NCT00529698; ISS T-001) and therapeutic (ClinicalTrials.gov Identifier: NCT00505401), double-blind, placebo-controlled phase I trials with the biologically active Tat were conducted in Italy, meeting both primary (safety) and secondary (immunogenicity) endpoints (Table 2) [172,173]. The Tat vaccine was safe and it did not induce virus replication in the HIV-infected volunteers, as indicated by the CD4 T cell counts preservation and the absence of significant plasma viremia rebounds. Antibody response to Tat lasted up to 5 years after vaccination, as determined in the long-term follow-up (ISS OBS P-001, ClinicalTrials.gov Identifier: NCT01024764) [172,173].

A phase I preventative trial with Tat and trimeric Env proteins was also conducted in Italy in volunteers at risk of HIV infection. The results indicate that vaccination is safe, immunogenic and generates strong antibody-dependent antiviral activities against both antigens [198].

**Table 2:** HIV-1 Tat vaccine clinical trials conducted at ISS.

Code (ClinicalTrials.gov Identifier)	Study	Country	Volunteers Enrolled	Reference
ISS P-001 (NCT00529698)	Phase I Preventive (Tat)	Italy	20	[172,173]
ISS OBS-001 (NCT01024764)	Phase I Preventive (Tat)	Italy	20	[172,173]
ISS P-002 (NCT01441193)	Phase I Preventive (Tat + Env)	Italy	11	Unpublished
ISS T-001 (NCT00505401)	Phase I Therapeutic (Tat)	Italy	27	[171,199]
ISS T-002 (NCT00751595)	Phase II Therapeutic (Tat)	Italy	168	[97,200]
ISS T-003 (NCT01513135)	Phase II Therapeutic (Tat)	South Africa	200	[174]
ISS T-002 EF-UP (NCT02118168)	Extended follow-up of ISS T-002	Italy	92	[201]
ISS T-003 EF-UP (NCT02712489)	Extended follow-up of ISS T-003	South Africa	179	Unpublished
	Total Volunteers		426	
	Vaccinated Volunteers		314	

### 8.2. Advanced (Phase II) Clinical Development

Therapeutic vaccination represents a shorter and cost-effective route to proof-of-efficacy, as compared to preventive trials [202]. As most PLWH do not develop, or have lost, antibodies to Tat, therapeutic vaccination with Tat is an excellent setting to verify the protective role of anti-Tat immunity, especially of anti-Tat Abs, traditionally easier to investigate, as compared to cell-mediated responses, which likely play a role but are more difficult to assess [169,203,204,205,206].

Therapeutic phase II trials for cART intensification were conducted in Italy and South Africa in patients on successful cART (Table 3). The Italian phase II study (ClinicalTrials.gov Identifier: NCT00751595) was an exploratory phase II open-label therapeutic trial, randomized on the different regimens utilized [97,200]. Both primary (immunogenicity) and secondary (safety) endpoints were met. No increase in virological biomarkers was observed. In particular, a reduction in immune activation and durable increases in CD4 T cells, B cells, NK cells and CD4 and CD8 central memory T cell subsets were observed in this trial but not in subjects on effective cART, negative for anti-Tat Ab and not immunized with the Tat vaccine enrolled in a parallel observational study conducted at the same clinical centers (ISS OBS T-002) (ClinicalTrials.gov Identifier: NCT01024556) [97,200]. Of note, results from the 8-year follow-up (ClinicalTrials.gov Identifier: NCT02118168) showed anti-Tat Abs’ persistence in more than 50% of volunteers, CD4 T cells’ increases durability and the progressive decline of HIV proviral DNA, which became undetectable in the blood of 34% of all vaccinees and in 48% of volunteers who had received three times the highest dose (30 μg) of the Tat vaccine [200,201]. These results indicate that the induction of anti-Tat immune responses intensifies cART efficacy and attacks the cART-resistant virus reservoir.

A confirmatory randomized, double-blind, placebo-controlled (randomization ratio 1:1), safety and immunogenicity phase II therapeutic trial (ISS T-003, ClinicalTrials.gov Identifier: NCT01513135) was then conducted in South Africa in 200 HIV-infected (C clade) anti-Tat Ab negative adults, virologically suppressed, with CD4 T cell counts ≥200 cells/mmc [174]. The vaccine was safe and induced durable and high titers of anti-Tat Abs that cross-recognize the Tat protein from different HIV clades and cross-neutralize both clade B and C HIV viruses. Cross-recognition and cross-neutralization correlated with the increase in CD4 T cell counts, a key target for cART intensification [174]. Of note, vaccination contained the VL rebound and maintained CD4 T cell counts above the baseline levels in subjects non-compliant to therapy as compared to (non-compliant) placebo, suggesting that the Tat vaccine intensification of cART may counterbalance incomplete adherence to treatment [174]. An extended follow-up study of this trial (ISS T-003 EF-UP, ClinicalTrials.gov identifier: NCT02712489) is underway. Overall, the Tat vaccine proves for the first time that cART can be intensified by therapeutic immunization and that proviral DNA load can be progressively lowered. Our data are consistent with those of Loret and colleagues, showing control of viremia upon cART interruption in volunteers vaccinated with a transactivation silent protein (Tat Oyi) [207]. In sharp contrast, no delay of virus rebound was observed upon vaccination with an immunodominant Tat peptide targeting a conserved B-cell epitope (Tat 4–12) and eliciting Abs recognizing all eight known Tat epitope variants [208], indicating that during immunization the whole Tat protein is preferable, as it triggers broad innate and adaptive immune responses, increasing the chances of the effective targeting of Tat.

## 9. Conclusions

A growing body of experimental evidence points to Tat as the critical factor in the virus life cycle, including the maintenance and replenishment of the virus reservoir, the major target of therapeutic vaccines in the cART era. In particular, Tat is one of the first HIV-1 proteins to be produced, as it is required for the expression of all other viral proteins. In addition, Tat is released extracellularly and may exert effects on both the virus and many cell types contributing to disease progression (before ART) and residual diseases (on effective ART). In this regard, emphasis should also be placed on the interactions of eTat with various opportunistic pathogens, which increase the incidence and severity of infections [102]. This is the case for Mycobacterium avium, whose infectivity is enhanced upon binding to Tat RGD via the integrin αvβ5, expressed on the cell [209], and for Leishmania in which Tat binding to LRP1/CD91 increases parasite uptake and intracellular growth in macrophages [210]. Thus, targeting Tat should irreversibly damage the HIV life cycle and hopefully control the infection (i.e., no residual disease or progression to disease) without therapy (functional cure).

The fact that, unlike all other HIV antigens, anti-Tat Abs do not develop in the majority of PLWH has certainly offered a unique opportunity to evaluate the impact of this response, providing epidemiological evidence to the rationale of targeting Tat. In the search for a functional cure, drugs inhibiting Tat expression are being tested in approaches aimed at blocking and locking HIV-1 gene expression. In particular, didehydro-Cortistatin A (dCA), a derivative of the natural steroidal alkaloid Corticostatin, is promising, as it sequesters intracellular Tat, blocking HIV-1 transcription [211], as well HIV-1 latency exit [212]. Of interest, dCA also binds eTat and inhibits eTat neurotoxicity in Tat transgenic mice [213], making it an attractive candidate to neutralize both intracellular and extracellular Tat activities.

The neutralization of Tat can also be achieved by vaccination with the Tat protein. Indeed, the results of preclinical and clinical studies support this view. Phase III therapeutic trials are needed to confirm the promising data and formally prove vaccine efficacy. Further, our data show that Tat binds the Env spikes displayed on the virion surface and that monkeys immunized with pre-mixed Tat and trimeric Env contained infection upon intrarectal challenge with a pathogenic simian-HIV chimeric virus (SHIV) preventing the virus from spreading beyond the rectum [82]. These data suggest that eTat also plays a key role in HIV-1 acquisition, providing the rationale to also include it in preventative vaccine approaches in association with Env, as also indicated by the generation of antibody-dependent antiviral activities in volunteers vaccinated with Tat and oligomeric Env combined.

## Figures and Tables

**Table 1:** Clinical benefits associated to the presence of anti-Tat antibodies.

Study Code	Volunteers Number	Status	Results	Potential Clinical Benefit
ISS OBS T-003 (NCT01029548) [192]	73	naïve to cART	Stable CD4 T cell counts and contained viral load in anti-Tat Ab positive individuals throughout the study (3 years) Persistently anti-Tat Ab positive: no progression or therapy initiation throughout the study (3 years) Transiently Ab positive: therapy initiation after 30 months Anti-Tat Ab negative: therapy initiation after 17 months	Prevention of progression
OBS-IFO [193]	29	starting cART	Faster and persistent virologic response to cART, in anti-Tat Ab positive as compared to anti-Tat Ab negative individuals	Improved time-to-response to therapy
ISS OBS T-002 (NCT01024556) [195]	127	on cART	CD4 T cell increase upon cART as compared to anti-Tat Ab neg individuals throughout the study (3 years)	Therapy intensification

Table reproduced and modified with permission from ref. [194].

**Table 3:** Tat vaccine phase II therapeutic clinical trials and observational follow-up studies conducted by ISS.

Trial Name	Registration Number	Description	Immune Responses	Virological Responses	References
ISS T-002 and ISS T-002 EF-UP (extended follow-up study)	NCT00751595 and NCT02118168	A 48-week randomized phase II, open-label, immunogenicity and safety trial in 168 anti-Tat negative HIV-1-infected cART-treated adult subjects. Volunteers intradermally received 7.5 or 30 µg of biologically active Tat, each dose given 3 or 5 times over 8 or 16 weeks. This 48-week study was followed by an extended (up to 5 years) follow-up.	Durable increase in CD4, B, and NK cell counts	HIV-1 DNA reduction 3 years after vaccination, which continued to decay in the 8 years of follow-up	[97,200,201]
			Reduction in immune activation		
			Restoration of functional CD4 and CD8 T cell subsets		
			Increased T-cell responses against Env and recall antigens		
ISS T-003 and ISS T-003 EF-UP (extended follow-up study)	NCT01513135 and NCT02712489	A 48-week randomized, double-blinded, placebo-controlled trial to evaluate immunogenicity and safety of B-clade Tat (30 μg) given intradermally, three times at 4-week intervals, in 200 HIV-infected adults on effective cART (randomized 1:1) with CD4 T cell counts ≥ 200 cells/µL. Study outcomes also included cross-clade anti-Tat antibodies, neutralization, CD4^+^ T-cell counts and therapy compliance. This 48-week study was followed by an extended (up to 5 years) follow-up.	Increase in CD4 T cell counts	In patients with detectable VL at Week 48, lower geometric mean levels in vaccinees	[174]
			Cross-clade Tat neutralizing antibodies		

## Data Availability

No new data were created or analyzed in this study. Data sharing is not applicable to this article.

## References

[1] Trickey A., Sabin C.A., Burkholder G., Crane H., Monforte A.D., Egger M., Gill M.J., Grabar S., Guest J.L., Jarrin I. (2023). Life expectancy after 2015 of adults with HIV on long-term antiretroviral therapy in Europe and North America: A collaborative analysis of cohort studies. Lancet HIV.

[2] Broyles L.N., Luo R., Boeras D., Vojnov L. (2023). The risk of sexual transmission of HIV in individuals with low-level HIV viraemia: A systematic review. Lancet.

[3] Aamer H.A., McClure J., Ko D., Maenza J., Collier A.C., Coombs R.W., Mullins J.I., Frenkel L.M. (2020). Cells producing residual viremia during antiretroviral treatment appear to contribute to rebound viremia following interruption of treatment. PLoS Pathog..

[4] Veenhuis R.T., Abreu C.M., Costa P.A.G., Ferreira E.A., Ratliff J., Pohlenz L., Shirk E.N., Rubin L.H., Blankson J.N., Gama L. (2023). Monocyte-derived macrophages contain persistent latent HIV reservoirs. Nat. Microbiol..

[5] Farhadian S.F., Lindenbaum O., Zhao J., Corley M.J., Im Y., Walsh H., Vecchio A., Garcia-Milian R., Chiarella J., Chintanaphol M. (2022). HIV viral transcription and immune perturbations in the CNS of people with HIV despite ART. JCI Insight.

[6] Asowata O.E., Singh A., Ngoepe A., Herbert N., Fardoos R., Reddy K., Zungu Y., Nene F., Mthabela N., Ramjit D. (2021). Irreversible depletion of intestinal CD4+ T cells is associated with T cell activation during chronic HIV infection. JCI Insight.

[7] Laspia M.F., Rice A.P., Mathews M.B. (1989). HIV-1 Tat protein increases transcriptional initiation and stabilizes elongation. Cell.

[8] Ensoli B., Barillari G., Salahuddin S.Z., Gallo R.C., Wong-Staal F. (1990). Tat protein of HIV-1 stimulates growth of cells derived from Kaposi’s sarcoma lesions of AIDS patients. Nature.

[9] Ensoli B., Gendelman R., Markham P., Fiorelli V., Colombini S., Raffeld M., Cafaro A., Chang H.K., Brady J.N., Gallo R.C. (1994). Synergy between basic fibroblast growth factor and HIV-1 Tat protein in induction of Kaposi’s sarcoma. Nature.

[10] Ensoli B., Buonaguro L., Barillari G., Fiorelli V., Gendelman R., Morgan R.A., Wingfield P., Gallo R.C. (1993). Release, uptake, and effects of extracellular HIV-1 Tat protein on cell growth and viral transactivation. J. Virol..

[11] Chang H.C., Samaniego F., Nair B.C., Buonaguro L., Ensoli B. (1997). HIV-1 Tat protein exits from cells via a leaderless secretory pathway and binds to extracellular matrix-associated heparan sulfate proteoglycans through its basic region. AIDS.

[12] Rayne F., Debaisieux S., Yezid H., Lin Y.-L., Mettling C., Konate K., Chazal N., Arold S.T., Pugnière M., Sanchez F. (2010). Phosphatidylinositol-(4,5)-bisphosphate enables efficient secretion of HIV-1 Tat by infected T-cells. EMBO J..

[13] Deeks S.G., Tracy R., Douek D.C. (2013). Systemic effects of inflammation on health during chronic HIV infection. Immunity.

[14] Feinberg M.B., Baltimore D., Frankel A.D. (1991). The role of Tat in the human immunodeficiency virus life cycle indicates a primary effect on transcriptional elongation. Proc. Natl. Acad. Sci. USA.

[15] Mueller N., Pasternak A.O., Klaver B., Cornelissen M., Berkhout B., Das A.T. (2018). The HIV-1 Tat Protein Enhances Splicing at the Major Splice Donor Site. J. Virol..

[16] D’Orso I., Frankel A.D. (2010). RNA-mediated displacement of an inhibitory snRNP complex activates transcription elongation. Nat. Struct. Mol. Biol..

[17] López-Huertas M.R., Callejas S., Abia D., Mateos E., Dopazo A., Alcami J., Coiras M. (2010). Modifications in host cell cytoskeleton structure and function mediated by intracellular HIV-1 Tat protein are greatly dependent on the second coding exon. Nucleic Acids Res..

[18] López-Huertas M.R., Mateos E., del Cojo M.S., Gómez-Esquer F., Díaz-Gil G., Rodríguez-Mora S., López J.A., Calvo E., López-Campos G., Alcamí J. (2013). The presence of HIV-1 Tat protein second exon delays fas protein-mediated apoptosis in CD4+ T lymphocytes: A potential mechanism for persistent viral production. J. Biol. Chem..

[19] Kukkonen S., Martinez-Viedma M.D.P., Kim N., Manrique M., Aldovini A. (2014). HIV-1 Tat second exon limits the extent of Tat-mediated modulation of interferon-stimulated genes in antigen presenting cells. Retrovirology.

[20] Howcroft T.K., Strebel K., Martin M.A., Singer D.S. (1993). Repression of MHC class I gene promoter activity by two-exon Tat of HIV. Science.

[21] Kanazawa S., Okamoto T., Peterlin B. (2000). Tat competes with CIITA for the binding to P-TEFb and blocks the expression of MHC class II genes in HIV infection. Immunity.

[22] Mele A.R., Marino J., Dampier W., Wigdahl B., Nonnemacher M.R. (2020). HIV-1 Tat Length: Comparative and Functional Considerations. Front. Microbiol..

[23] Epie N., Ammosova T., Sapir T., Voloshin Y., Lane W.S., Turner W., Reiner O., Nekhai S. (2005). HIV-1 Tat interacts with LIS1 protein. Retrovirology.

[24] Chen D., Wang M., Zhou S., Zhou Q. (2002). HIV-1 Tat targets microtubules to induce apoptosis, a process promoted by the pro-apoptotic Bcl-2 relative Bim. EMBO J..

[25] Huo L., Li D., Sun L., Liu M., Shi X., Sun X., Li J., Dong B., Dong X., Zhou J. (2010). Tat acetylation regulates its actions on microtubule dynamics and apoptosis in T lymphocytes. J. Pathol..

[26] Wei P., Garber M.E., Fang S.-M., Fischer W.H., Jones K.A. (1998). A novel CDK9-associated C-type cyclin interacts directly with HIV-1 Tat and mediates its high-affinity, loop-specific binding to TAR RNA. Cell.

[27] Berkhout B., Silverman R.H., Jeang K.-T. (1989). Tat trans-activates the human immunodeficiency virus through a nascent RNA target. Cell.

[28] Gotora P.T., van der Sluis R., Williams M.E. (2023). HIV-1 Tat amino acid residues that influence Tat-TAR binding affinity: A scoping review. BMC Infect. Dis..

[29] Tyagi M., Rusnati M., Presta M., Giacca M. (2001). Internalization of HIV-1 tat requires cell surface heparan sulfate proteoglycans. J. Biol. Chem..

[30] Ruiz A.P., Ajasin D.O., Ramasamy S., DesMarais V., Eugenin E.A., Prasad V.R. (2019). A Naturally Occurring Polymorphism in the HIV-1 Tat Basic Domain Inhibits Uptake by Bystander Cells and Leads to Reduced Neuroinflammation. Sci. Rep..

[31] Gump J.M., June R.K., Dowdy S.F. (2010). Revised role of glycosaminoglycans in TAT protein transduction domain-mediated cellular transduction. J. Biol. Chem..

[32] King J., Eugenin E., Buckner C., Berman J. (2006). HIV tat and neurotoxicity. Microbes Infect..

[33] Brake D.A., Debouck C., Biesecker G. (1990). Identification of an Arg-Gly-Asp (RGD) cell adhesion site in human immunodeficiency virus type 1 transactivation protein, tat. J. Cell Biol..

[34] Barillari G., Gendelman R., Gallo R.C., Ensoli B. (1993). The Tat protein of human immunodeficiency virus type 1, a growth factor for AIDS Kaposi sarcoma and cytokine-activated vascular cells, induces adhesion of the same cell types by using integrin receptors recognizing the RGD amino acid sequence. Proc. Natl. Acad. Sci. USA.

[35] Neuveut C., Jeang K.T. (1996). Recombinant human immunodeficiency virus type 1 genomes with tat unconstrained by overlapping reading frames reveal residues in Tat important for replication in tissue culture. J. Virol..

[36] Neuveut C., Scoggins R.M., Camerini D., Markham R.B., Jeang K.-T. (2003). Requirement for the second coding exon of Tat in the optimal replication of macrophage-tropic HIV-1. J. Biomed. Sci..

[37] Rodríguez-Mora S., Mateos E., Moran M., Martín M., López J.A., Calvo E., Terrón M.C., Luque D., Muriaux D., Alcamí J. (2015). Intracellular expression of Tat alters mitochondrial functions in T cells: A potential mechanism to understand mitochondrial damage during HIV-1 replication. Retrovirology.

[38] Wu Y., Marsh J.W. (2001). Selective transcription and modulation of resting T cell activity by preintegrated HIV DNA. Science.

[39] Chertova E., Chertov O., Coren L.V., Roser J.D., Trubey C.M., Bess J.W., Sowder R.C., Barsov E., Hood B.L., Fisher R.J. (2006). Proteomic and biochemical analysis of purified human immunodeficiency virus type 1 produced from infected monocyte-derived macrophages. J. Virol..

[40] Schatz M., Marty L., Ounadjela C., Tong P.B.V., Cardace I., Mettling C., Milhiet P.-E., Costa L., Godefroy C., Pugnière M. (2023). A Tripartite Complex HIV-1 Tat-Cyclophilin A-Capsid Protein Enables Tat Encapsidation That Is Required for HIV-1 Infectivity. J. Virol..

[41] Harrich D., Ulich C., García-Martínez L.F., Gaynor R.B. (1997). Tat is required for efficient HIV-1 reverse transcription. EMBO J..

[42] Mele A.R., Marino J., Chen K., Pirrone V., Janetopoulos C., Wigdahl B., Klase Z., Nonnemacher M.R. (2018). Defining the molecular mechanisms of HIV-1 Tat secretion: PtdIns(4,5)P_2_ at the epicenter. Traffic.

[43] Ghanam R.H., Eastep G.N., Saad J.S. (2023). Structural Insights into the Mechanism of HIV-1 Tat Secretion from the Plasma Membrane. J. Mol. Biol..

[44] Jost M., Simpson F., Kavran J.M., Lemmon M.A., Schmid S.L. (1998). Phosphatidylinositol-4,5-bisphosphate is required for endocytic coated vesicle formation. Curr. Biol..

[45] Botelho R.J., Teruel M., Dierckman R., Anderson R., Wells A., York J.D., Meyer T., Grinstein S. (2000). Localized biphasic changes in phosphatidylinositol-4,5-bisphosphate at sites of phagocytosis. J. Cell Biol..

[46] Debaisieux S., Lachambre S., Gross A., Mettling C., Besteiro S., Yezid H., Henaff D., Chopard C., Mesnard J.-M., Beaumelle B. (2015). HIV-1 Tat inhibits phagocytosis by preventing the recruitment of Cdc42 to the phagocytic cup. Nat. Commun..

[47] Tryoen-Tóth P., Chasserot-Golaz S., Tu A., Gherib P., Bader M.-F., Beaumelle B., Vitale N. (2013). HIV-1 Tat protein inhibits neurosecretion by binding to phosphatidylinositol 4,5-bisphosphate. J. Cell Sci..

[48] Harwig A., Jongejan A., van Kampen A.H.C., Berkhout B., Das A.T. (2016). Tat-dependent production of an HIV-1 TAR-encoded miRNA-like small RNA. Nucleic Acids Res..

[49] Sampey G.C., Saifuddin M., Schwab A., Barclay R., Punya S., Chung M.-C., Hakami R.M., Zadeh M.A., Lepene B., Klase Z.A. (2016). Exosomes from HIV-1-infected Cells Stimulate Production of Pro-inflammatory Cytokines through Trans-activating Response (TAR) RNA. J. Biol. Chem..

[50] Chen L., Feng Z., Yue H., Bazdar D., Mbonye U., Zender C., Harding C.V., Bruggeman L., Karn J., Sieg S.F. (2018). Exosomes derived from HIV-1-infected cells promote growth and progression of cancer via HIV TAR RNA. Nat. Commun..

[51] Chettimada S., Lorenz D.R., Misra V., Dillon S.T., Reeves R.K., Manickam C., Morgello S., Kirk G.D., Mehta S.H., Gabuzda D. (2018). Exosome markers associated with immune activation and oxidative stress in HIV patients on antiretroviral therapy. Sci. Rep..

[52] Johnson T.P., Patel K., Johnson K.R., Maric D., Calabresi P.A., Hasbun R., Nath A. (2013). Induction of IL-17 and nonclassical T-cell activation by HIV-Tat protein. Proc. Natl. Acad. Sci. USA.

[53] Dahl V., Lee E., Peterson J., Spudich S.S., Leppla I., Sinclair E., Fuchs D., Palmer S., Price R.W. (2011). Raltegravir treatment intensification does not alter cerebrospinal fluid HIV-1 infection or immunoactivation in subjects on suppressive therapy. J. Infect. Dis..

[54] Xiao H., Neuveut C., Tiffany H.L., Benkirane M., Rich E.A., Murphy P.M., Jeang K.-T. (2000). Selective CXCR4 antagonism by Tat: Implications for *in vivo* expansion of coreceptor use by HIV-1. Proc. Natl. Acad. Sci. USA.

[55] Poggi A., Zocchi M.R. (2006). HIV-1 Tat triggers TGF-beta production and NK cell apoptosis that is prevented by pertussis toxin B. J. Immunol. Res..

[56] Westendorp M.O., Frank R., Ochsenbauer C., Stricker K., Dhein J., Walczak H., Debating K.-M., Krammer P.H. (1995). Sensitization of T cells to CD95-mediated apoptosis by HIV-1 Tat and gp120. Nature.

[57] Marchiò S., Alfano M., Primo L., Gramaglia D., Butini L., Gennero L., De Vivo E., Arap W., Giacca M., Pasqualini R. (2005). Cell surface-associated Tat modulates HIV-1 infection and spreading through a specific interaction with gp120 viral envelope protein. Blood.

[58] Weinberger L.S., Burnett J.C., Toettcher J.E., Arkin A.P., Schaffer D.V. (2005). Stochastic gene expression in a lentiviral positive-feedback loop: HIV-1 Tat fluctuations drive phenotypic diversity. Cell.

[59] Buonaguro L., Buonaguro F.M., Giraldo G., Ensoli B. (1994). The human immunodeficiency virus type 1 Tat protein transactivates tumor necrosis factor beta gene expression through a TAR-like structure. J. Virol..

[60] Nappi F., Chiozzini C., Bordignon V., Borsetti A., Bellino S., Cippitelli M., Barillari G., Caputo A., Tyagi M., Giacca M. (2009). Immobilized HIV-1 Tat protein promotes gene transfer via a transactivation-independent mechanism which requires binding of Tat to viral particles. J. Gene Med..

[61] Zauli G., Gibellini D., Celeghini C., Mischiati C., Bassini A., La Placa M., Capitani S. (1996). Pleiotropic effects of immobilized versus soluble recombinant HIV-1 Tat protein on CD3-mediated activation, induction of apoptosis, and HIV-1 long terminal repeat transactivation in purified CD4+ T lymphocytes. J. Immunol..

[62] Ott M., Emiliani S., Van Lint C., Herbein G., Lovett J., Chirmule N., McCloskey T., Pahwa S., Verdin E. (1997). Immune hyperactivation of HIV-1-infected T cells mediated by Tat and the CD28 pathway. Science.

[63] Li C.J., Ueda Y., Shi B., Borodyansky L., Huang L., Li Y.-Z., Pardee A.B. (1997). Tat protein induces self-perpetuating permissivity for productive HIV-1 infection. Proc. Natl. Acad. Sci. USA.

[64] Gavioli R., Gallerani E., Fortini C., Fabris M., Bottoni A., Canella A., Bonaccorsi A., Marastoni M., Micheletti F., Cafaro A. (2004). HIV-1 tat protein modulates the generation of cytotoxic T cell epitopes by modifying proteasome composition and enzymatic activity. J. Immunol..

[65] Campbell G.R., Loret E.P. (2009). What does the structure-function relationship of the HIV-1 Tat protein teach us about developing an AIDS vaccine?. Retrovirology.

[66] Fanales-Belasio E., Moretti S., Nappi F., Barillari G., Micheletti F., Cafaro A., Ensoli B. (2002). Native HIV-1 Tat protein targets monocyte-derived dendritic cells and enhances their maturation, function, and antigen-specific T cell responses. J. Immunol..

[67] Fanales-Belasio E., Moretti S., Fiorelli V., Tripiciano A., Cossut M.R.P., Scoglio A., Collacchi B., Nappi F., Macchia I., Bellino S. (2009). HIV-1 Tat Addresses Dendritic Cells to Induce a Predominant Th1-Type Adaptive Immune Response That Appears Prevalent in the Asymptomatic Stage of Infection. J. Immunol..

[68] Li J.C.-B., Yim H.C.-H., Lau A.S. (2010). Role of HIV-1 Tat in AIDS pathogenesis: Its effects on cytokine dysregulation and contributions to the pathogenesis of opportunistic infection. AIDS.

[69] Chopard C., Tong P.B.V., Tóth P., Schatz M., Yezid H., Debaisieux S., Mettling C., Gross A., Pugnière M., Tu A. (2018). Cyclophilin A enables specific HIV-1 Tat palmitoylation and accumulation in uninfected cells. Nat. Commun..

[70] Baggaley R.F., White R.G., Boily M.-C. (2010). HIV transmission risk through anal intercourse: Systematic review, meta-analysis and implications for HIV prevention. Leuk. Res..

[71] Vittinghoff E., Scheer S., O’Malley P., Colfax G., Holmberg S.D., Buchbinder S.P. (1999). Combination antiretroviral therapy and recent declines in AIDS incidence and mortality. J. Infect. Dis..

[72] Jin F., Jansson J., Law M., Prestage G.P., Zablotska I., Imrie J.C., Kippax S.C., Kaldor J.M., Grulich A.E., Wilson D.P. (2010). Per-contact probability of HIV transmission in homosexual men in Sydney in the era of HAART. AIDS.

[73] Boily M.-C., Baggaley R.F., Wang L., Masse B., White R.G., Hayes R.J., Alary M. (2009). Heterosexual risk of HIV-1 infection per sexual act: Systematic review and meta-analysis of observational studies. Lancet Infect. Dis..

[74] Ward H., Rönn M. (2010). Contribution of sexually transmitted infections to the sexual transmission of HIV. Curr. Opin. HIV AIDS.

[75] Quinn T.C., Wawer M.J., Sewankambo N., Serwadda D., Li C., Wabwire-Mangen F., Meehan M.O., Lutalo T., Gray R.H. (2000). Viral load and heterosexual transmission of human immunodeficiency virus type 1. Rakai Project Study Group. N. Engl. J. Med..

[76] Baeten J.M., Kahle E., Lingappa J.R., Coombs R.W., Delany-Moretlwe S., Nakku-Joloba E., Mugo N.R., Wald A., Corey L., Donnell D. (2011). Genital HIV-1 RNA predicts risk of heterosexual HIV-1 transmission. Sci. Transl. Med..

[77] Wawer M.J., Gray R.H., Sewankambo N.K., Serwadda D., Li X., Laeyendecker O., Kiwanuka N., Kigozi G., Kiddugavu M., Lutalo T. (2005). Rates of HIV-1 transmission per coital act, by stage of HIV-1 infection, in Rakai, Uganda. J. Infect. Dis..

[78] Hollingsworth T.D., Anderson R.M., Fraser C. (2008). HIV-1 transmission, by stage of infection. J. Infect. Dis..

[79] Caputo V., Libera M., Sisti S., Giuliani B., Diotti R.A., Criscuolo E. (2023). The initial interplay between HIV and mucosal innate immunity. Front. Immunol..

[80] Kariuki S.M., Selhorst P., Ariën K.K., Dorfman J.R. (2017). The HIV-1 transmission bottleneck. Retrovirology.

[81] Oberle C.S., Joos B., Rusert P., Campbell N.K., Beauparlant D., Kuster H., Weber J., Schenkel C.D., Scherrer A.U., The Swiss HIV Cohort Study (SHCS) (2016). Tracing HIV-1 transmission: Envelope traits of HIV-1 transmitter and recipient pairs. Retrovirology.

[82] Monini P., Cafaro A., Srivastava I.K., Moretti S., Sharma V.A., Andreini C., Chiozzini C., Ferrantelli F., Cossut M.R.P., Tripiciano A. (2012). HIV-1 Tat Promotes Integrin-Mediated HIV Transmission to Dendritic Cells by Binding Env Spikes and Competes Neutralization by Anti-HIV Abs. PLoS ONE.

[83] Ward A.B., Wilson I.A. (2015). Insights into the trimeric HIV-1 envelope glycoprotein structure. Trends Biochem. Sci..

[84] Huet T., Dazza M.-C., Brun-Vézinet F., Roelants G.E., Wain-Hobson S. (1989). A highly defective HIV-1 strain isolated from a healthy Gabonese individual presenting an atypical Western blot. AIDS.

[85] Anderson E.M., Simonetti F.R., Gorelick R.J., Hill S., Gouzoulis M.A., Bell J., Rehm C., Pérez L., Boritz E., Wu X. (2020). Dynamic Shifts in the HIV Proviral Landscape During Long Term Combination Antiretroviral Therapy: Implications for Persistence and Control of HIV Infections. Viruses.

[86] Botha J.C., Demirov D., Gordijn C., Katusiime M.G., Bale M.J., Wu X., Wells D., Hughes S.H., Cotton M.F., Mellors J.W. (2023). The largest HIV-1-infected T cell clones in children on long-term combination antiretroviral therapy contain solo LTRs. mBio.

[87] Lorenzo-Redondo R., Fryer H.R., Bedford T., Kim E.Y., Archer J., Kosakovsky Pond S.L.K., Chung Y.S., Penugonda S., Chipman J.G., Fletcher C.V. (2016). Persistent HIV-1 replication maintains the tissue reservoir during therapy. Nature.

[88] Fletcher C.V., Staskus K., Wietgrefe S.W., Rothenberger M., Reilly C., Chipman J.G., Beilman G.J., Khoruts A., Thorkelson A., Schmidt T.E. (2014). Persistent HIV-1 replication is associated with lower antiretroviral drug concentrations in lymphatic tissues. Proc. Natl. Acad. Sci. USA.

[89] Sigal A., Kim J.T., Balazs A.B., Dekel E., Mayo A., Milo R., Baltimore D. (2011). Cell-to-cell spread of HIV permits ongoing replication despite antiretroviral therapy. Nature.

[90] Martinez-Picado J., Zurakowski R., Buzón M.J., Stevenson M. (2018). Episomal HIV-1 DNA and its relationship to other markers of HIV-1 persistence. Retrovirology.

[91] Lassen K.G., Ramyar K.X., Bailey J.R., Zhou Y., Siliciano R.F. (2006). Nuclear retention of multiply spliced HIV-1 RNA in resting CD4+ T cells. PLoS Pathog..

[92] Wietgrefe S.W., Anderson J., Duan L., Southern P.J., Zuck P., Wu G., Howell B.J., Reilly C., Kroon E., Chottanapund S. (2023). Initial productive and latent HIV infections originate in vivo by infection of resting T cells. J. Clin. Investig..

[93] Weinberger L.S., Dar R.D., Simpson M.L. (2008). Transient-mediated fate determination in a transcriptional circuit of HIV. Nat. Genet..

[94] Razooky B.S., Pai A., Aull K., Rouzine I.M., Weinberger L.S. (2015). A Hardwired HIV Latency Program. Cell.

[95] Zerbato J.M., Purves H.V., Lewin S.R., Rasmussen T.A. (2019). Between a shock and a hard place: Challenges and developments in HIV latency reversal. Curr. Opin. Virol..

[96] Moranguinho I., Valente S.T. (2020). Block-And-Lock: New Horizons for a Cure for HIV-1. Viruses.

[97] Ensoli B., Bellino S., Tripiciano A., Longo O., Francavilla V., Marcotullio S., Cafaro A., Picconi O., Paniccia G., Scoglio A. (2010). Therapeutic immunization with HIV-1 Tat reduces immune activation and loss of regulatory T-cells and improves immune function in subjects on HAART. PLoS ONE.

[98] Mediouni S., Darque A., Baillat G., Ravaux I., Dhiver C., Tissot-Dupont H., Mokhtari M., Moreau H., Tamalet C., Brunet C. (2012). Antiretroviral therapy does not block the secretion of the human immunodeficiency virus tat protein. Infect. Disord. Drug Targets.

[99] Nicoli F., Gallerani E., Sforza F., Finessi V., Chachage M., Geldmacher C., Cafaro A., Ensoli B., Caputo A., Gavioli R. (2018). The HIV-1 Tat protein affects human CD4+ T-cell programing and activation, and favors the differentiation of naïve CD4+ T cells. AIDS.

[100] Shan L., Deng K., Gao H., Xing S., Capoferri A.A., Durand C.M., Rabi S.A., Laird G.M., Kim M., Hosmane N.N. (2017). Transcriptional reprogramming during effector-to-memory transition renders CD4+ T cells permissive for latent HIV-1 infection. Immunity.

[101] Zauli G., Gibellini D., Caputo A., Bassini A., Negrini M., Monne M., Mazzoni M., Capitani S. (1995). The human immunodeficiency virus type-1 Tat protein upregulates Bcl-2 gene expression in Jurkat T-cell lines and primary peripheral blood mononuclear cells. Blood.

[102] Ensoli B., Moretti S., Borsetti A., Maggiorella M.T., Buttò S., Picconi O., Tripiciano A., Sgadari C., Monini P., Cafaro A. (2021). New insights into pathogenesis point to HIV-1 Tat as a key vaccine target. Arch. Virol..

[103] Péloponèse J.M., Collette Y., Grégoire C., Bailly C., Campèse D., Meurs E.F., Olive D., Loret E.P. (1999). Full peptide synthesis purification and characterization of six Tat variants. Differences observed between HIV-1 isolates from Africa and other continents. J. Biol. Chem..

[104] Ludwig B.S., Kessler H., Kossatz S., Reuning U. (2021). RGD-Binding Integrins Revisited: How Recently Discovered Functions and Novel Synthetic Ligands (Re-)Shape an Ever-Evolving Field. Cancers.

[105] Barillari G., Sgadari C., Fiorelli V., Samaniego F., Colombini S., Manzari V., Modesti A., Nair B.C., Cafaro A., Stürzl M. (1999). The Tat protein of human immunodeficiency virus type-1 promotes vascular cell growth and locomotion by engaging the a5b3 integrins and by mobilizing sequestered basic fibroblast growth factor. Blood.

[106] Samaniego F., Markham P.D., Gendelman R., Gallo R.C., Ensoli B. (1997). Inflammatory cytokines induce endothelial cells to produce and release basic fibroblast growth factor and to promote Kaposi’s sarcoma-like lesions in nude mice. J. Immunol..

[107] Cafaro A., Barillari G., Moretti S., Palladino C., Tripiciano A., Falchi M., Picconi O., Cossut M.R.P., Campagna M., Arancio A. (2021). HIV-1 Tat Protein Enters Dysfunctional Endothelial Cells via Integrins and Renders Them Permissive to Virus Replication. Int. J. Mol. Sci..

[108] Savill J., Dransfield I., Hogg N., Haslett C. (1990). Vitronectin receptor-mediated phagocytosis of cells undergoing apoptosis. Nature.

[109] Antonov A.S., Antonova G.N., Munn D.H., Mivechi N., Lucas R., Catravas J.D., Verin A.D. (2010). ααvβ3 Integrin Regulates Macrophage Inflammatory Responses via PI3 Kinase/Akt-Dependent NF-κB Activation. J. Cell. Physiol..

[110] Kumawat A.K., Yu C., Mann E.A., Schridde A., Finnemann S.C., Mowat A.M. (2018). Expression and characterization of αvβ5 integrin on intestinal macrophages. Eur. J. Immunol..

[111] Rubartelli A., Poggi A., Zocchi M.R. (1997). The selective engulfment of apoptotic bodies by dendritic cells is mediated by the alpha(v)beta3 integrin and requires intracellular and extracellular calcium. Eur. J. Immunol..

[112] Albert M.L., Pearce S.F.A., Francisco L.M., Sauter B., Roy P., Silverstein R.L., Bhardwaj N. (1998). Immature Dendritic Cells Phagocytose Apoptotic Cells via αvβ5 and CD36, and Cross-present Antigens to Cytotoxic T Lymphocytes. J. Exp. Med..

[113] Barillari G., Albonici L., Incerpi S., Bogetto L., Pistritto G., Volpi A., Ensoli B., Manzari V. (2001). Inflammatory cytokines stimulate vascular smooth muscle cells locomotion and growth by enhancing alpha5beta1 integrin expression and function. Atherosclerosis.

[114] Labus J., Wöltje K., Stolte K.N., Häckel S., Kim K.S., Hildmann A., Danker K. (2018). IL-1_ promotes transendothelial migration of PBMCs by upregulation of the FN/α5β1 sig-nalling pathway in immortalised human brain microvascular endothelial cells. Exp. Cell Res..

[115] Albini A., Barillari G., Benelli R., Gallo R.C., Ensoli B. (1995). Angiogenic properties of human immunodeficiency virus type 1 Tat protein. Proc. Natl. Acad. Sci. USA.

[116] Fiorelli V., Barillari G., Toschi E., Sgadari C., Monini P., Sturzl M., Ensoli B. (1999). Interferon-γ induces endothelial cells to proliferate and to invade the extracellular matrix in response to HIV-1 Tat. J. Immunol..

[117] Bussolino F., Mitola S., Serini G., Barillari G., Ensoli B. (2001). Interactions between endothelial cells and HIV-1. Int. J. Biochem. Cell Biol..

[118] Urbinati C., Bugatti A., Giacca M., Schlaepfer D., Presta M., Rusnati M. (2005). αvβ3 integrin-dependent activation of focal adhesion kinase mediatesNF-_B activation and motogenic activity by HIV-1 Tat in endothelial cells. J. Cell Sci..

[119] Urbinati C., Mitola S., Tanghetti E., Kumar C., Waltenberger J., Ribatti D., Presta M., Rusnati M. (2005). Integrin αvβ_3_as a Target for Blocking HIV-1 Tat-Induced Endothelial Cell Activation In Vitro and Angiogenesis In Vivo. Arter. Thromb. Vasc. Biol..

[120] Sforza F., Nicoli F., Gallerani E., Finessi V., Reali E., Cafaro A., Caputo A., Ensoli B., Gavioli R. (2014). HIV-1 Tat affects the programming and functionality of human CD8^+^ T cells by modulating the expression of T-box transcription factors. AIDS.

[121] Ensoli B., Sgadari C., Barillari G., Sirianni M.C., Stürzl M., Monini P. (2001). Biology of Kaposi’s Sarcoma. Eur. J. Cancer.

[122] Barillari G., Sgadari C., Palladino C., Gendelman R., Caputo A., Morris C.B., Nair B.C., Markham P., Nel A., Stürzl M. (1999). Inflammatory cytokines synergize with the HIV-1 Tat protein to promote angiogenesis and Kaposi’s sarcoma via induction of basic fibroblast growth factor and the alpha v beta 3 integrin. J. Immunol..

[123] Prakash O., Tang Z.-Y., He Y.-E., Ali M.S., Coleman R., Gill J., Farr G., Samaniego F. (2000). Human Kaposi’s sarcoma cell-mediated tumorigenesis in human immunodeficiency type 1 Tat-expressing transgenic mice. J. Natl. Cancer Inst..

[124] Lämmermann T., Bader B.L., Monkley S.J., Worbs T., Wedlich-Söldner R., Hirsch K., Keller M., Förster R., Critchley D.R., Fässler R. (2008). Rapid leukocyte migration by integrin-independent flowing and squeezing. Nature.

[125] Huttenlocher A., Horwitz A.R. (2011). Integrins in Cell Migration. Cold Spring Harb. Perspect. Biol..

[126] Yin C., Heit B. (2021). Cellular Responses to the Efferocytosis of Apoptotic Cells. Front. Immunol..

[127] Acharya A.P., Dolgova N.V., Moore N.M., Xia C.-Q., Clare-Salzler M.J., Becker M.L., Gallant N.D., Keselowsky B.G. (2010). The modulation of dendritic cell integrin binding and activation by RGD-peptide density gradient substrates. Biomaterials.

[128] Carbone F.R., Bevan M.J. (1990). Class I-restricted processing and presentation of exogenous cell-associated antigen in vivo. J. Exp. Med..

[129] Sigal L.J., Crotty S., Andino R., Rock K.L. (1999). Cytotoxic T-cell immunity to virus-infected non-haematopoietic cells requires presentation of exogenous antigen. Nature.

[130] Baxter A.E., Russell R.A., Duncan C.J., Moore M.D., Willberg C.B., Pablos J.L., Finzi A., Kaufmann D.E., Ochsenbauer C., Kappes J.C. (2014). Macrophage Infection via Selective Capture of HIV-1-Infected CD4^+^ T Cells. Cell Host Microbe.

[131] Mortier A., Gouwy M., Van Damme J., Proost P., Struyf S. (2016). CD26/dipeptidylpeptidase IV—Chemokine interactions: Double-edged regulation of inflammation and tumor biology. J. Leukoc. Biol..

[132] Klemann C., Wagner L., Stephan M., von Hörsten S. (2016). Cut to the chase: A review of CD26/dipeptidyl peptidase-4’s (DPP4) entanglement in the immune system. Clin. Exp. Immunol..

[133] Reinhold D., Bank U., Bühling F., Lendeckel U., Faust J., Neubert K., Ansorge S. (1997). Inhibitors of dipeptidyl peptidase IV induce secretion of transforming growth factor-beta 1 in PWM-stimulated PBMC and T cells. Immunology.

[134] Preller V., Gerber A., Wrenger S., Togni M., Marguet D., Tadje J., Lendeckel U., Röcken C., Faust J., Neubert K. (2007). TGF-beta1-mediated control of central nervous system inflammation and autoimmunity through the inhibitory receptor CD26. J. Immunol..

[135] Ploquin M.J., Casrouge A., Madec Y., Noël N., Jacquelin B., Huot N., Duffy D., Jochems S.P., Micci L., Lécuroux C. (2018). Systemic DPP4 activity is reduced during primary HIV-1 infection and is associated with intestinal RORC^+^CD4^+^cell levels: A surrogate marker candidate of HIV-induced intestinal damage. J. Int. AIDS Soc..

[136] Theron A.J., Anderson R., Rossouw T.M., Steel H.C. (2017). The Role of Transforming Growth Factor Beta-1 in the Progression of HIV/AIDS and Development of Non-AIDS-Defining Fibrotic Disorders. Front. Immunol..

[137] Gutheil W.G., Subramanyam M., Flentke G.R., Sanford D.G., Munoz E., Huber B.T., Bachovchin W.W. (1994). Human immunodeficiency virus 1 Tat binds to dipeptidyl aminopeptidase IV (CD26): A possible mechanism for Tat’s immunosuppressive activity. Proc. Natl. Acad. Sci. USA.

[138] Subramanyam M., Gutheil W.G., Bachovchin W.W., Huber B.T. (1993). Mechanism of HIV-1 Tat induced inhibition of antigen-specific T cell responsiveness. J. Immunol..

[139] Wrenger S., Reinhold D., Hoffmann T., Kraft M., Frank R., Faust J., Neubert K., Ansorge S. (1996). The N-terminal X-X-Pro sequence of the HIV-1 Tat protein is important for the inhibition of dipeptidyl peptidase IV (DP IV/CD26) and the suppression of mitogen-induced proliferation of human T cells. FEBS Lett..

[140] Zauli G., Davis B.R., Re M.C., Visani G., Furlini G., La Placa M. (1992). tat protein stimulates production of transforming growth factor-beta 1 by marrow macrophages: A potential mechanism for human immunodeficiency virus-1-induced hematopoietic suppression. Blood.

[141] Schmitz T., Underwood R., Khiroya R., Bachovchin W.W., Huber B.T. (1996). Potentiation of the immune response in HIV-1+ individuals. J. Clin. Investig..

[142] Lafrenie R.M., Wahl L.M., Epstein J.S., Hewlett I.K., Yamada K.M., Dhawan S. (1996). HIV-1-Tat modulates the function of monocytes and alters their interactions with mi-crovessel endothelial cells. A mechanism of HIV pathogenesis. J. Immunol..

[143] Lafrenie R.M., Wahl L.M., Epstein J.S., Hewlett I.K., Yamada K.M., Dhawan S. (1996). HIV-1-Tat protein promotes chemotaxis and invasive behavior by monocytes. J. Immunol..

[144] Albini A., Benelli R., Giunciuglio D., Cai T., Mariani G., Ferrini S., Noonan D.M. (1998). Identification of a novel domain of HIV tat involved in monocyte chemotaxis. J. Biol. Chem..

[145] Albini A., Ferrini S., Benelli R., Sforzini S., Giunciuglio D., Aluigi M.G., Proudfoot A.E.I., Alouani S., Wells T.N.C., Mariani G. (1998). HIV-1 Tat protein mimicry of chemokines. Proc. Natl. Acad. Sci. USA.

[146] Huang L., Bosch I., Hofmann W., Sodroski J., Pardee A.B. (1998). Tat protein induces human immunodeficiency virus type 1 (HIV-1) coreceptors and promotes infection with both macrophage-tropic and T-lymphotropic HIV-1 strains. J. Virol..

[147] Campbell G.R., Loret E.P., Spector S.A. (2010). HIV-1 clade B Tat, but not clade C Tat, increases X4 HIV-1 entry into resting but not activated CD4+ T cells. J. Biol. Chem..

[148] Packard T.A., Schwarzer R., Herzig E., Rao D., Luo X., Egedal J.H., Hsiao F., Widera M., Hultquist J.F., Grimmett Z.W. (2022). CCL2: A Chemokine Potentially Promoting Early Seeding of the Latent HIV Reservoir. mBio.

[149] Covino D.A., Sabbatucci M., Fantuzzi L. (2016). The CCL2/CCR2 Axis in the Pathogenesis of HIV-1 Infection: A New Cellular Target for Therapy?. Curr. Drug Targets.

[150] Chu H.X., Arumugam T.V., Gelderblom M., Magnus T., Drummond G.R., Sobey C.G. (2014). Role of CCR2 in Inflammatory conditions of the central nervous system. J. Cereb. Blood Flow Metab..

[151] Dyer D.P., Medina-Ruiz L., Bartolini R., Schuette F., Hughes C.E., Pallas K., Vidler F., Macleod M.K.L., Kelly C.J., Lee K.M. (2019). Chemokine Receptor Redundancy and Specificity Are Context Dependent. Immunity.

[152] Fantuzzi L., Tagliamonte M., Gauzzi M.C., Lopalco L. (2019). Dual CCR5/CCR2 targeting: Opportunities for the cure of complex disorders. Cell. Mol. Life Sci..

[153] Deeks S.G., Lewin S.R., Havlir D.V. (2013). The end of AIDS: HIV infection as a chronic disease. Lancet.

[154] Lerner A.M., Eisinger R.W., Fauci A.S. (2020). Comorbidities in Persons With HIV: The Lingering Challenge. JAMA.

[155] Zhang H., Yang K., Chen F., Liu Q., Ni J., Cao W., Hua Y., He F., Liu Z., Li L. (2022). Role of the CCL2-CCR2 axis in cardiovascular disease: Pathogenesis and clinical implications. Front. Immunol..

[156] Bose S., Cho J. (2013). Role of chemokine CCL2 and its receptor CCR2 in neurodegenerative diseases. Arch. Pharmacal Res..

[157] Curzytek K., Leśkiewicz M. (2021). Targeting the CCL2-CCR2 axis in depressive disorders. Pharmacol. Rep..

[158] Zhu S., Liu M., Bennett S., Wang Z., Pfleger K.D.G., Xu J. (2021). The molecular structure and role of CCL2 (MCP-1) and C-C chemokine receptor CCR2 in skeletal biology and diseases. J. Cell. Physiol..

[159] She S., Ren L., Chen P., Wang M., Chen D., Wang Y., Chen H. (2022). Functional Roles of Chemokine Receptor CCR2 and Its Ligands in Liver Disease. Front. Immunol..

[160] Xu M., Wang Y., Xia R., Wei Y., Wei X. (2021). Role of the CCL2-CCR2 signalling axis in cancer: Mechanisms and therapeutic targeting. Cell Prolif..

[161] O’Connor T., Borsig L., Heikenwalder M. (2015). CCL2-CCR2 Signaling in Disease Pathogenesis. Endocr. Metab. Immune Disord. Drug Targets.

[162] Kashi V.P., Jacob R.A., Paul S., Nayak K., Satish B., Swaminathan S., Satish K.S., Ranga U. (2009). HIV-1 Tat-specific IgG antibodies in high-responders target a B-cell epitope in the cysteine-rich domain and block extracellular Tat efficiently. Vaccine.

[163] Lederman M.M., Funderburg N.T., Sekaly R.P., Klatt N.R., Hunt P.W. (2013). Residual immune dysregulation syndrome in treated HIV infection. Adv. Immunol..

[164] Planès R., Bahraoui E. (2013). HIV-1 Tat protein induces the production of IDO in human monocyte derived-dendritic cells through a direct mechanism: Effect on T cells proliferation. PLoS ONE.

[165] Ben Haij N., Planès R., Leghmari K., Serrero M., Delobel P., Izopet J., BenMohamed L., Bahraoui E. (2015). HIV-1 Tat Protein Induces Production of Proinflammatory Cytokines by Human Dendritic Cells and Monocytes/Macrophages through Engagement of TLR4-MD2-CD14 Complex and Activation of NF-κB Pathway. PLoS ONE.

[166] Planès R., BenMohamed L., Leghmari K., Delobel P., Izopet J., Bahraoui E. (2014). HIV-1 Tat protein induces PD-L1 (B7-H1) expression on dendritic cells through tumor necrosis factor alpha- and toll-like receptor 4-mediated mechanisms. J. Virol..

[167] Caputo A., Brocca-Cofano E., Castaldello A., Voltan R., Gavioli R., Srivastava I.K., Barnett S.W., Cafaro A., Ensoli B. (2008). Characterization of immune responses elicited in mice by intranasal co-immunization with HIV-1 Tat, gp140 ΔV2Env and/or SIV Gag proteins and the nontoxicogenic heat-labile Escherichia coli enterotoxin. Vaccine.

[168] Gavioli R., Cellini S., Castaldello A., Voltan R., Gallerani E., Gagliardoni F., Fortini C., Cofano E.B., Triulzi C., Cafaro A. (2008). The Tat protein broadens T cell responses directed to the HIV-1 antigens Gag and Env: Implications for the design of new vaccination strategies against AIDS. Vaccine.

[169] Cafaro A., Caputo A., Fracasso C., Maggiorella M.T., Goletti D., Baroncelli S., Pace M., Sernicola L., Koanga-Mogtomo M.L., Betti M. (1999). Control of SHIV-89.6P-infection of cynomolgus monkeys by HIV-1 Tat protein vaccine. Nat. Med..

[170] Cafaro A., Bellino S., Titti F., Maggiorella M.T., Sernicola L., Wiseman R.W., Venzon D., Karl J.A., O’Connor D., Monini P. (2010). Impact of viral dose and major histocompatibility complex class IB haplotype on viral outcome in mauritian cynomolgus monkeys vaccinated with Tat upon challenge with simian/human immunodeficiency virus SHIV89.6P. J. Virol..

[171] Ensoli B., Fiorelli V., Ensoli F., Lazzarin A., Visintini R., Narciso P., Di Carlo A., Monini P., Magnani M., Garaci E. (2008). The therapeutic phase I trial of the recombinant native HIV-1 Tat protein. AIDS.

[172] Ensoli B., Fiorelli V., Ensoli F., Lazzarin A., Visintini R., Narciso P., Di Carlo A., Tripiciano A., Longo O., Bellino S. (2009). The preventive phase I trial with the HIV-1 Tat-based vaccine. Vaccine.

[173] Bellino S., Francavilla V., Longo O., Tripiciano A., Paniccia G., Arancio A., Fiorelli V., Scoglio A., Collacchi B., Campagna M. (2009). Parallel conduction of the phase I preventive and therapeutic trials based on the Tat vaccine candidate. Rev. Recent Clin. Trials.

[174] Ensoli B., Nchabeleng M., Ensoli F., Tripiciano A., Bellino S., Picconi O., Sgadari C., Longo O., Tavoschi L., SMU-MeCRU Study Group (2016). HIV-Tat immunization induces cross-clade neutralizing antibodies and CD4^+^ T cell increases in antiretroviral-treated South African volunteers: A randomized phase II clinical trial. Retrovirology.

[175] Lillis A.P., Van Duyn L.B., Murphy-Ullrich J.E., Strickland D.K., Sarhan M., Land W.G., Tonnus W., Hugo C.P., Linkermann A., Hopkins P.N. (2008). LDL receptor-related protein 1: Unique tissue-specific functions revealed by selective gene knockout studies. Physiol. Rev..

[176] Sizova O., John L.S., Ma Q., Molldrem J.J. (2023). Multi-faceted role of LRP1 in the immune system. Front. Immunol..

[177] Talme T., Bergdahl E., Sundqvist K.G. (2014). Regulation of T-lymphocyte motility, adhesion and de-adhesion by a cell surface mechanism directed by low density lipoprotein receptor-related protein 1 and endogenous thrombospondin-1. Immunology.

[178] Sundqvist K.G. (2018). T Cell Co-Stimulation: Inhibition of Immunosuppression?. Front. Immunol..

[179] Stebbing J., Gazzard B., Portsmouth S., Gotch F., Kim L., Bower M., Mandalia S., Binder R., Srivastava P., Patterson S. (2003). Disease-associated dendritic cells respond to disease-specific antigens through the common heat shock protein receptor. Blood.

[180] Kebba A., Stebbing J., Rowland S., Ingram R., Agaba J., Patterson S., Kaleebu P., Imami N., Gotch F. (2005). Expression of the common heat-shock protein receptor CD91 is increased on monocytes of exposed yet HIV-1-seronegative subjects. J. Leukoc. Biol..

[181] Liu Y., Jones M., Hingtgen C.M., Bu G., Laribee N., Tanzi R.E., Moir R.D., Nath A., He J.J. (2000). Uptake of HIV-1 tat protein mediated by low-density lipoprotein receptor-related protein disrupts the neuronal metabolic balance of the receptor ligands. Nat. Med..

[182] Liu Z., Christensson M., Forslöw A., De Meester I., Sundqvist K.-G. (2009). A CD26-controlled cell surface cascade for regulation of T cell motility and chemokine signals. J. Immunol..

[183] McCloskey T.W., Ott M., Tribble E., Khan S.A., Teichberg S., Paul M.O., Pahwa S., Verdin E., Chirmule N. (1997). Dual role of HIV Tat in regulation of apoptosis in T cells. J. Immunol..

[184] Chandrasekar A.P., Cummins N.W., Badley A.D. (2019). The Role of the BCL-2 Family of Proteins in HIV-1 Pathogenesis and Persistence. Clin. Microbiol. Rev..

[185] Krone W.J., Debouck C., Epstein L.G., Heutink P., Meloen R., Goudsmit J. (1988). Natural antibodies to HIV-tat epitopes and expression of HIV-1 genes in vivo. J. Med. Virol..

[186] Reiss P., Lange J.M., de Ronde A., de Wolf F., Dekker J., Debouck C., Goudsmit J. (1990). Speed of progression to AIDS and degree of antibody response to accessory gene products of HIV-1. J. Med. Virol..

[187] Re M.C., Furlini G., Vignoli M., Ramazzotti E., Roderigo G., De Rosa V., Zauli G., Lolli S., Capitani S., La Placa M. (1995). Effect of antibody to HIV-1 Tat protein on viral replication in vitro and progression of HIV-1 disease in vivo. J. Acquir. Immune Defic. Syndr. Hum. Retrovirol..

[188] Re M.C., Vignoli M., Furlini G., Gibellini D., Colangeli V., Vitone F., La Placa M. (2001). Antibodies against full-length Tat protein and some low-molecular-weight Tat-peptides correlate with low or undetectable viral load in HIV-1 seropositive patients. J. Clin. Virol..

[189] Zagury J.F., Sill A., Blattner W., Lachgar A., Le Buanec H., Richardson M., Rappaport J., Hendel H., Bizzini B., Gringeri A. (1998). Antibodies to the HIV-1 Tat protein correlated with nonprogression to AIDS: A rationale for the use of Tat toxoid as an HIV-1 vaccine. J. Hum. Virol..

[190] Richardson M.W., Mirchandani J., Duong J., Grimaldo S., Kocieda V., Hendel H., Khalili K., Zagury J.-F., Rappaport J. (2003). Antibodies to Tat and Vpr in the GRIV cohort: Differential association with maintenance of long-term non-progression status in HIV-1 infection. Biomed. Pharmacother..

[191] Rezza G., Fiorelli V., Dorrucci M., Ciccozzi M., Tripiciano A., Scoglio A., Collacchi B., Ruiz-Alvarez M., Giannetto C., Caputo A. (2005). The presence of anti-Tat antibodies is predictive of long-term nonprogression to AIDS or severe immunodeficiency: Findings in a cohort of HIV-1 seroconverters. J. Infect. Dis..

[192] Bellino S., Tripiciano A., Picconi O., Francavilla V., Longo O., Sgadari C., Paniccia G., Arancio A., Angarano G., Ladisa N. (2014). The presence of anti-Tat antibodies in HIV-infected individuals is associated with containment of CD4^+^ T-cell decay and viral load, and with delay of disease progression: Results of a 3-year cohort study. Retrovirology.

[193] Cafaro A., Tripiciano A., Sgadari C., Bellino S., Picconi O., Longo O., Francavilla V., Buttò S., Titti F., Monini P. (2015). Development of a novel AIDS vaccine: The HIV-1 transactivator of transcription protein vaccine. Expert Opin. Biol. Ther..

[194] Cafaro A., Tripiciano A., Picconi O., Sgadari C., Moretti S., Buttò S., Monini P., Ensoli B. (2019). Anti-Tat Immunity in HIV-1 Infection: Effects of Naturally Occurring and Vaccine-Induced Antibodies Against Tat on the Course of the Disease. Vaccines.

[195] Tripiciano A., Picconi O., Moretti S., Sgadari C., Cafaro A., Francavilla V., Arancio A., Paniccia G., Campagna M., Pavone-Cossut M.R. (2021). Anti-Tat immunity defines CD4+ T-cell dynamics in people living with HIV on long-term cART. EBioMedicine.

[196] Maggiorella M.T., Baroncelli S., Michelini Z., Fanales-Belasio E., Moretti S., Sernicola L., Cara A., Negri D.R., Buttò S., Fiorelli V. (2004). Long-term protection against SHIV89.6P replication in HIV-1 Tat vaccinated cynomolgus monkeys. Vaccine.

[197] Borsetti A., Baroncelli S., Maggiorella M.T., Moretti S., Fanales-Belasio E., Sernicola L., Tripiciano A., Macchia I., Michelini Z., Belli R. (2009). Containment of infection in tat vaccinated monkeys after rechallenge with a higher dose of SHIV89.6P(cy243). Viral Immunol..

[198] Ensoli B. (2024). National HIV/AIDS Research Center.

[199] Longo O., Tripiciano A., Fiorelli V., Bellino S., Scoglio A., Collacchi B., Alvarez M.J.R., Francavilla V., Arancio A., Paniccia G. (2009). Phase I therapeutic trial of the HIV-1 Tat protein and long term follow-up. Vaccine.

[200] Ensoli F., Cafaro A., Casabianca A., Tripiciano A., Bellino S., Longo O., Francavilla V., Picconi O., Sgadari C., Moretti S. (2015). HIV-1 Tat immunization restores immune homeostasis and attacks the HAART-resistant blood HIV DNA: Results of a randomized phase II exploratory clinical trial. Retrovirology.

[201] Sgadari C., Monini P., Tripiciano A., Picconi O., Casabianca A., Orlandi C., Moretti S., Francavilla V., Arancio A., Paniccia G. (2019). Continued Decay of HIV Proviral DNA Upon Vaccination With HIV-1 Tat of Subjects on Long-Term ART: An 8-Year Follow-Up Study. Front. Immunol..

[202] Ensoli B., Cafaro A., Monini P., Emarcotullio S., Ensoli F. (2014). Challenges in HIV Vaccine Research for Treatment and Prevention. Front. Immunol..

[203] van Baalen C.A., Huisman R.C., Klein M.R., Gruters R.A., Miedema F., Geretti A.M., Osterhaus A.D., Pontesilli O., de Wolf F. (1997). Human immunodeficiency virus type 1 Rev- and Tat-specific cytotoxic T lymphocyte frequencies inversely correlate with rapid progression to AIDS. J. Gen. Virol..

[204] Addo M.M., Altfeld M., Rosenberg E.S., Eldridge R.L., Philips M.N., Habeeb K., Khatri A., Brander C., Robbins G.K., Mazzara G.P. (2001). The HIV-1 regulatory proteins Tat and Rev are frequently targeted by cytotoxic T lymphocytes derived from HIV-1-infected individuals. Proc. Natl. Acad. Sci. USA.

[205] Cao J., McNevin J., Malhotra U., McElrath M.J. (2003). Evolution of CD8+ T cell immunity and viral escape following acute HIV-1 infection. J. Immunol..

[206] Jones N.A., Wei X., Flower D.R., Wong M., Michor F., Saag M.S., Hahn B.H., Nowak M.A., Shaw G.M., Borrow P. (2004). Determinants of human immunodeficiency virus type 1 escape from the primary CD8+ cytotoxic T lymphocyte response. J. Exp. Med..

[207] Loret E.P., Darque A., Jouve E., Loret E.A., Nicolino-Brunet C., Morange S., Castanier E., Casanova J., Caloustian C., Bornet C. (2016). Intradermal injection of a Tat Oyi-based therapeutic HIV vaccine reduces of 1.5 log copies/mL the HIV RNA rebound median and no HIV DNA rebound following cART interruption in a phase I/II randomized controlled clinical trial. Retrovirology.

[208] Goldstein G., Damiano E., Donikyan M., Pasha M., Beckwith E., Chicca J. (2012). HIV-1 Tat B-cell epitope vaccination was ineffectual in preventing viral rebound after ART cessation: HIV rebound with current ART appears to be due to infection with new endogenous founder virus and not to resurgence of pre-existing Tat-dependent viremia. Hum. Vaccines Immunother..

[209] Denis M. (1994). Tat protein from HIV-1 binds to Mycobacterium avium via a bacterial integrin. Effects on extracellular and intracellular growth. J. Immunol..

[210] Lodge R., Ouellet M., Barat C., Andreani G., Kumar P., Tremblay M.J. (2012). HIV-1 promotes intake of Leishmania parasites by enhancing phosphatidylserine-mediated, CD91/LRP-1-dependent phagocytosis in human macrophages. PLoS ONE.

[211] Mousseau G., Clementz M.A., Bakeman W.N., Nagarsheth N., Cameron M., Shi J., Baran P., Fromentin R., Chomont N., Valente S.T. (2012). An analog of the natural steroidal alkaloid cortistatin A potently suppresses Tat-dependent HIV transcription. Cell Host Microbe.

[212] Mousseau G., Kessing C.F., Fromentin R., Trautmann L., Chomont N., Valente S.T. (2015). The Tat Inhibitor Didehydro-Cortistatin A Prevents HIV-1 Reactivation from Latency. mBio.

[213] Mediouni S., Jablonski J., Paris J.J., Clementz M.A., Thenin-Houssier S., McLaughlin J.P., Valente S.T. (2015). Didehydro-cortistatin A inhibits HIV-1 Tat mediated neuroinflammation and prevents potentiation of cocaine reward in Tat transgenic mice. Curr. HIV Res..

